# Complete Mapping of Substrate Translocation Highlights the Role of LeuT N-terminal Segment in Regulating Transport Cycle

**DOI:** 10.1371/journal.pcbi.1003879

**Published:** 2014-10-09

**Authors:** Mary Hongying Cheng, Ivet Bahar

**Affiliations:** Department of Computational and Systems Biology, School of Medicine, University of Pittsburgh, Pittsburgh, United States of America; Max Planck Institute for Biophysical Chemistry, Germany

## Abstract

Neurotransmitter: sodium symporters (NSSs) regulate neuronal signal transmission by clearing excess neurotransmitters from the synapse, assisted by the co-transport of sodium ions. Extensive structural data have been collected in recent years for several members of the NSS family, which opened the way to structure-based studies for a mechanistic understanding of substrate transport. Leucine transporter (LeuT), a bacterial orthologue, has been broadly adopted as a prototype in these studies. This goal has been elusive, however, due to the complex interplay of global and local events as well as missing structural data on LeuT N-terminal segment. We provide here for the first time a comprehensive description of the molecular events leading to substrate/Na^+^ release to the postsynaptic cell, including the structure and dynamics of the N-terminal segment using a combination of molecular simulations. Substrate and Na^+^-release follows an influx of water molecules into the substrate/Na^+^-binding pocket accompanied by concerted rearrangements of transmembrane helices. A redistribution of salt bridges and cation-π interactions at the N-terminal segment prompts substrate release. Significantly, substrate release is followed by the closure of the intracellular gate and a global reconfiguration back to outward-facing state to resume the transport cycle. Two minimally hydrated intermediates, not structurally resolved to date, are identified: one, substrate-bound, stabilized during the passage from outward- to inward-facing state (*holo-occluded*), and another, substrate-free, along the reverse transition (*apo-occluded*).

## Author Summary

Bacterial leucine transporter (LeuT) belongs to neurotransmitter:sodium symporter (NSS) family. Its human orthologs include dopamine transporter and serotonin transporter. Malfunction of NSS members has been implicated in neurological diseases, hence the significance of elucidating their mechanism of function as clinically relevant drug targets. NSSs co-transport substrates (neurotransmitters or amino acids) and sodium ions across the cell membrane via alternating access to extracellular and intracellular media, which enables the uptake of substrate and ions from the extracellular region and their release to the intracellular region. Despite significant progress in elucidating the structure and function of NSS family members, their mechanism of function and the role of their N-terminal segment exposed to the cell interior remain elusive. Here, we provide for the first time a full-atomic time-resolved description of the complete transport cycle of LeuT using multiscale simulations. Two major findings are (i) elucidation of the structure and dynamics of the N-terminal segment which helps in mediating substrate and cation release and resuming the transport cycle, and (ii) determination of the structures of two minimally hydrated intermediates occluded to both extracellular and intracellular environments.

## Introduction

Neurotransmitter:sodium symporters (NSSs) play a vital role in regulating neurotransmission and preventing neurotoxicity by timely uptake of their substrate (neurotransmitters such as dopamine, serotonin, norepinephrine or GABA, or small molecules and amino acids) from the synapse. Transport of substrate takes place against its 10^6^-fold concentration increase in the intracellular (IC) environment compared to extracellular (EC) [1], enabled by the symport of Na^+^ ions down their electrochemical gradient. Several NSS family members such as dopamine transporter (DAT) and serotonin transporter (SERT) are targets for addictive drugs and antidepressants [2].

Sodium-coupled neurotransmitter transporters are generally accepted to function via the classical alternating access mechanism [1], [3]: they alternate between outward-facing (OF) and inward-facing (IF) states that expose their substrate-binding pocket to the EC and IC environments, for substrate uptake and release, respectively. Each state assumes in turn two substates, *open* (*o*) and *closed* (*c*), defined by the local reconfigurations of structural elements serving as EC/IC gates. Substrate transport thus involves both *global* transitions (OF↔IF) between the two states as well as *local* transitions (*c ↔ o*) within each state [4]. The transport cycle may thus be postulated to proceed via a series of transitions 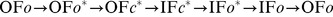
(I)where the asterisk designates substrate/Na^+^-bound form. The successive steps are: substrate binding to OF open state (OF*o* → OFo*); EC gate closure (OFo* → OFc*); transition to IF state (OFc* → IFc*); IC gate opening (IFc* → IFo*); release of substrate (IFo* → IF*o*); and transition back to OF*o* (IF*o* → OF*o*). However, this scheme involves conformers (IFc* and IFo*) that have not been experimentally resolved to date. Additionally, the possible stabilization of other intermediates during the transport cycle is not yet established, nor do we have a clear understanding of time-resolved atomic events that enable the transitions between those states.

Leucine transporter (LeuT) from *Aquifex aeolicus* became a prototype for structure-based studies of NSS functioning, as the first crystallographically resolved member of the family [5]–[7]. Crystallographic structures have been resolved for OF*o* [6], OFo* [7], OFc* [5] (in the presence of two Na^+^ ions and a Leu) and IF*o* [6] states. Despite considerable progress in establishing NSS structure-function relations [1], [8], many aspects of NSS transport remain to be understood. First, the N-terminal segment has not been resolved in the IF state. This segment has been pointed out, in eukaryotes, to affect IC gating [9], drug modulation [10], and DAT endocytosis [11]. Elucidating not only the structure but also the dynamics of the N-terminal segment is a significant goal. Second, the OF ↔ IF transition is beyond the reach of conventional molecular dynamics (cMD) simulations; cMD is limited to microseconds for such systems composed of ∼10^5^ atoms (with explicit lipid and water molecules), even with the use of cutting-edge technologies [12]. Not surprisingly, computational studies of LeuT and its homologues [13]–[20] have mainly focused on local events (reviewed in [21]). Third, it remains to be established whether the transport cycle proceeds via occluded (or other) intermediates.

Multiscale methodologies that combine conventional simulations for visualizing local events [14]–[18], [20] and accelerated simulations for assessing collective motions [4], [13], [19] albeit at low resolution, present useful tools for exploring coupled global and local events. We adopted such an approach here: we performed a series of conventional (cMD), targeted (tMD) [13] and accelerated MD (aMD) [22] simulations (**Table 1**).

**Table 1:** Summary of simulated systems and processes, simulation types and durations, and initial states.

Run #	Observed process(a)		Run Identifier	Duration (ns)	Initial conformer	RMSD (Å)(b)
*1*	OFc*	Fluctuations in the neighborhood of the OFc* state	cMD1_OFc*	30	OFc* crystal	0.0 (2A65)
*2*			cMD2_OFc*	30		0.0 (2A65)
*3*	OFc* - - -> IF*o*	Triggering of conformational change away from OFc* toward IFo*	tMD1_OFc*	10	OFc* from simulations (c)	0.8 (2A65)
*4*			tMD2_OFc*	20		
*5*			tMD3_OFc*	10	OFc* equilibrated	1.2 (2A65)
*6*	OFc*→ *holo-occluded*	Transition to, and stabilization of, *holo -occluded* state	aMD_*holo*	94	6.8 ns of *run* 3	2.1 (2A65)
*7*			cMD_*holo*	94		
*8*	*holo-occluded* → IFo*→ IF*o*	Opening of the IC vestibule, release of Ala	cMD1_*holo* →IF*o*	233	7.4 ns of *run* 3	1.5 (3TT3)
*9*			cMD2_*holo* →IF*o*	93		1.5 (3TT3)
*10*	IFo*→IF*o*	Release of Ala and Na^+^ to IC region, stabilization of IF*o*	cMD1_IFo*→IF*o*	91	9.0 ns of *run* 5	1.2 (3TT3)
*11*			cMD2_IFo*→IF*o*	80	end of *run* 4	0.8 (3TT3)
*12*	IF*o*	Fluctuations near IF*o* crystal structure	cMD1_IF*o*	30	no N-terminal res R5-T10	0.0 (3TT3)
*13*			cMD2_IF*o*	30		0.0 (3TT3)
*14*		Association of the N-terminus with either TM7 orTM5, irrespective of starting N-terminal conformation	cMD3_IF*o*	30	N-terminus in *conformation 1*	0.0 (3TT3)
*15*			cMD4_IF*o*	30		0.0 (3TT3)
*16*			cMD5_IF*o*	30	N-terminus in *conformation 2*	0.0 (3TT3)
*17*			cMD6_IF*o*	30		0.0 (3TT3)
*18*	IFo*→IF*o*→ *apo-occluded*	Transition to *apo- occluded* state	cMD3_IFo* →*apo*	192	8.0 ns of *run 3*	1.2 (3TT3)
*19*	IF*o*→ *apo-occluded*		cMD4_ IF*o*→*apo*	60	42 ns of *run 18*	1.9 (3TT3)

(a) OF and IF refer to the outward- and inward-facing states; *o* and *c* refer to the *open* or *closed* conformations of the EC or IC gates in either state; asterisk is appended when there is a bound substrate.

(b) backbone RMSD with respect to the crystal structure written in parentheses (2A65 and 3TT3 are the respective PDB ids for OFc* and IF*o*).

(c) OFc* conformation generated in *run 7* of ref 4.

**Figure 1:**
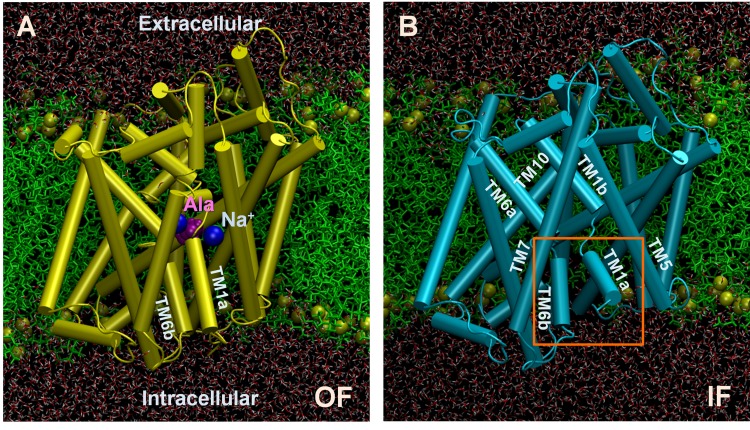
Outward-facing (OF) and inward-facing (IF) states of LeuT, displayed in explicit lipid and water molecules. The panels display the MD set ups of (**A**) LeuT OFc* (PDB: 2A65; *orange*) and (**B)** IF*o* (PDB: 3TT3; *cyan*) structures embedded into POPC lipid bilayer (*green*) and solvated by 0.1 M NaCl (not shown) solution. POPC phosphorus atoms are shown in *tan* spheres, water molecules in *red lines*. The *blue* spheres in (**A**) represent the two Na^+^ ions immobilized in the crystal structure. The bound Leu in the crystal structure is replaced by Ala (*purple*) in the simulations. Helices labeled in (**B**), including the broken helices TM1a-b and TM6a-b, exhibit notable reorientations.

In our previous work [4], we examined substrate-binding events and succeeding EC gate closure, OF*o* → OFo* → OFc*. Here we focus on substrate release, starting from the OFc* state (**Figure 1A**), and examine the sequence of events all the way to the inward-facing open state, IF*o* (**Figure 1B**), and back transition toward OF*o*. We focus in particular on the structure and dynamics of the N-terminal segment during substrate release and reconfiguration back to an *apo-occluded* form. As in our previous study [4], we use alanine as substrate because LeuT transports Ala more efficiently than Leu [7].

The present simulations provide for the first time a full-atomic description of the structure, dynamics and functional role of LeuT N-terminal segment in the nano-to-microseconds time regime. They also elucidate the conformers that are temporarily stabilized during the transport cycle. Substrate binding is observed to disrupt the tight packing between the transmembrane (TM) helices TM1, TM5, TM6 and TM8 and to trigger their concerted tiltings, facilitated by influx of water molecules to the binding site. Strikingly, a well-defined redistribution of salt bridges and cation-π interactions at the N-terminal segment closes back the IC gate following the release of substrate, which further drives the transition back toward the OF state. Based on these observations, we propose the N-terminal segment to serve as a regulatory element that controls the IC gate and restores the transporter structure back to its OF state to resume the transport cycle. Another important result is the elucidation of two intermediate structures, both occluded to IC and EC regions, one *holo*, the other *apo*, that have not (yet) been experimentally observed for LeuT.

## Results

### Overview of the method of approach and simulations

We adopted a multiscale approach that combines cMD, tMD and aMD simulations, in accord with the methodology that proved useful in a recent study [4]. The cMD and aMD simulations were initiated with a variety of conformers to ensure broad coverage of the conformational space, including snapshots from short tMD runs that triggered the transition towards the IF*o* state. We have intentionally selected to perform short (10–20 ns) tMD runs, followed by long (∼100 ns) unbiased MD simulations, so as to avoid artificial conformations that would be enforced by tMD. In line with traditional approaches, the targeted force was applied to the protein backbone only, and then the conventional MD runs would let the side chains reorient and relax before exploring the conformational space and possible stabilization of intermediates. Details on the simulation protocols and parameters are provided in the Methods.

**Table 1** provides a summary of the simulations. The runs permitted us to explore the vicinity of the OFc* state (*runs 1* and *2*); trigger the reconfiguration toward the IF state (*runs 3–5*); identify a new intermediate, ligand/ion-bound, occluded to both EC and IC environments, called *holo-occluded* (*runs 6–8*); and visualize the release of substrate and ions starting from *holo-occluded* (*runs 8–9*; **Movie S1**) or IFo* (*runs 8–11* and *18*), the conformational fluctuations in the IF*o* state (*runs 12–17*), and the transition from IF*o* into *apo-occluded* (*runs 18 and 19*; **Movie S2**). The present simulations, together with those presented earlier [4] on substrate- and ion-binding events, permit us to map for the first time the *complete* sequence of events taking place during LeuT transport cycle.

### OF ↔ IF transition involves intermediate states with distinctive hydration patterns, helix packing properties and N-terminal interactions

**Figure 2:**
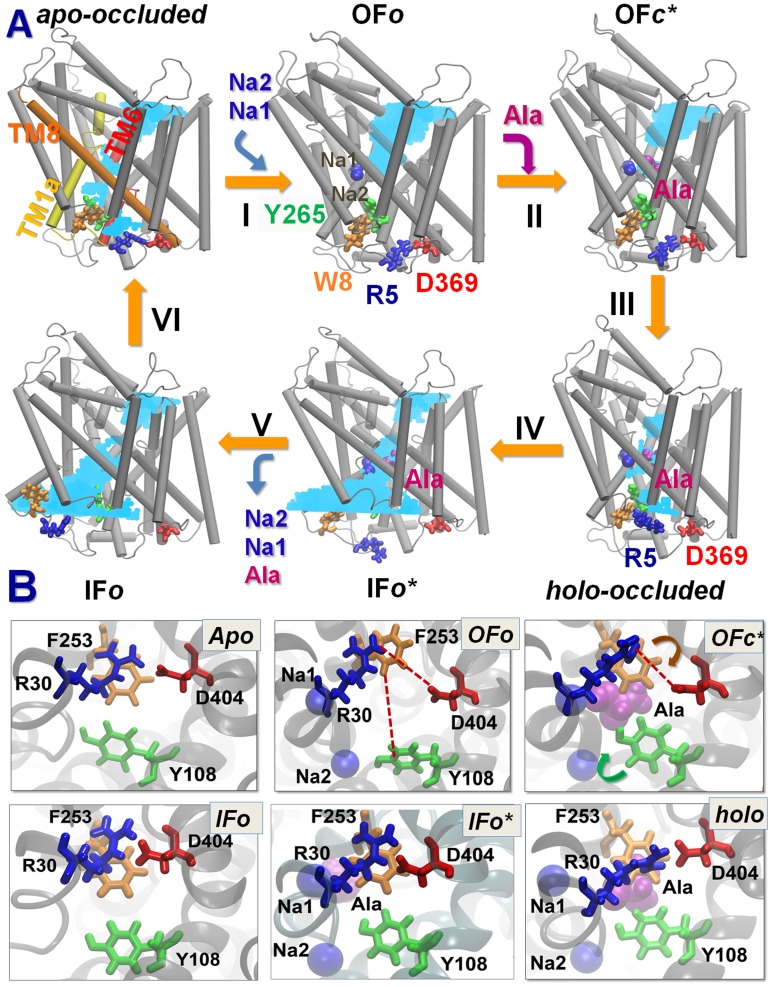
Conformational states visited by LeuT during its transport cycle and corresponding hydration patterns and changes in interactions at IC and EC gates. (**A**) Six states, labeled, are distinguished, including three newly determined ones: *holo-occluded*, inward-facing substrate-bound open (IFo*), and *apo-occluded*. The association/dissociation of the two putative IC gating pairs, R5-D369 and W8-Y268-Y265 (shown in *licorice*), distinguishes the OF and IF states, along with changes in TM1 and TM6 orientations. Hydrated regions are indicated by *blue* shaded areas. (**B**) Two EC gates R30-D404 and F253-Y108 exhibit *closed* or *open* (indicated by *red dashed line*) conformations depending on the LeuT state. At least one of the EC gates is closed in all states, except in OF*o*. In *holo-occluded* and *apo-occluded* states, the substrate binding site is practically occluded to both EC and IC environments, with at least one EC gate and one IC gate being closed concurrently.

Our first aim was to explore the states that have not been crystallographically resolved to date, IFc* and IFo* where the IC gates are *c*losed and *o*pen, respectively, or intermediates which might be temporarily stabilized along the transport cycle. Simulations revealed the stabilization of six states (**Figure 2A**) along the transport cycle: 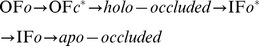
(II)which includes three newly elucidated states: (i) a minimally-hydrated *holo-occluded* state occluded to both EC and IC regions (in lieu of IFc* state), (ii) the IFo* state, and (iii) another intermediate state, *apo-occluded*, during the back transition from IF to OF in substrate/ion-free state. The OFo* conformer was hardly detected, presumably due to allosteric coupling between substrate binding and EC gate closing. We note that the experimentally resolved OFo* structure is trapped in an inactive state via binding of the competitive inhibitor Trp. Binding of Ala, a natural substrate, on the other hand, cooperatively stimulated EC gate closure [4].

The six states were distinguished by three major criteria: First, they exhibit distinctive hydration patterns (*cyan shades* in **Figure 2A**). In the OF state, the EC-exposed vestibule is hydrated, while the IC-facing region is completely dehydrated. In the IF state, on the other hand, the IC-exposed vestibule is hydrated while the EC-exposed region shows small hydration, separated from the IC vestibule by a dehydrated region such that no leakage of water takes place. The substrate/ion binding pocket is minimally hydrated and occluded to both EC and IC environments in both *holo-occluded* and *apo-occluded* states.

Second, the states have distinctive inter-helical packing properties, which were quantified by evaluating the center-of-mass (CoM) distances between pairs of TM helical segments selected to provide discriminative descriptions: TM1a-TM6b on the IC side, and TM1b-TM10 and TM6a-TM10 (based on extracellular half of TM10) on the EC side (see **Figure 1B**). The former provides a measure of the opening of the IC vestibule [23], and the latter two, that of the EC vestibule [23]. In line with previous work [23], **Table 2** clearly shows the qualitative and quantitative differences in the interhelical packing characteristics of the states. Note the similarity in interhelical packing between *holo-* and *apo-occluded* forms.

**Table 2:** Interhelical distances at different states observed in simulations and in the crystal structures.

C^α^ distances between TM helices* (Å)	*OFo/OFo* *		*OFc* *		*Holo-occluded*		*IFo* * */IFo*		*Apo-occluded*	
	Simulations	Crystal	Simulations	Crystal	Simulations	Crystal	Simulations	Crystal	Simulations	Crystal
**TM1b-TM10**	18.0±0.6	18.6	16.8±0.3	16.2	15.8±0.4	N/A	14.3±0.4	13.8	14.5±0.4	N/A
**TM6a-TM10**	16.8±0.4	16.6	13.8±0.3	13.8	12.5±0.4	N/A	12.2±0.4	11.5	13.2±0.3	N/A
**TM1a-TM6b**	11.6±0.2	11.4	11.7±0.2	11.6	12.8±0.4	N/A	20.5±2.0	22.6	12.2±0.3	N/A

* TM1a (R11 to A22), TM1b (L25 to A35), TM6a (G242 to L255), TM6b (F259 to Y268), and TM10 EC half (K398 to V412) C^α^ atoms were used for the calculations of the center of mass. Results for OF*o*/OFo* were taken from our previous study (ref 4); results for OFc* were averaged based on *runs 1* to *2*; for *holo-occluded* state, equilibrated conformers in *runs 6* and *7* were used; for IF*o*/IFo*, results were averaged based on *runs 8* to *17*; for *apo-occluded* state, equilibrated conformers in *runs 18* and *19 were* taken.

Third, a series of amino acids at the N-terminus (and in particular R5, E6 and W8 which are conserved in LeuT, DAT, and SERT) were distinguished by the redistributions of interactions with residues at the IC-exposed ends of TM6b and TM8 (Y265-Y268 and D369, respectively) suggesting a regulation of the transition to IF*o* state, and back to the uptake-ready state (**Figure 2A**). Likewise, EC-gating amino acid pairs, R30-D404 and F253-Y108, exhibited distinctive interaction patterns in these states (**Figure 2B**).

In the following subsections, we will elaborate on all these three aspects, starting from the structural features of the *holo-occluded* state and the observed mechanism of substrate release. These will be followed by detailed descriptions of the events at the N-terminus, which mediate the transition from OFc* to IF*o* and opening of the IC pore, and the return to uptake-ready state after Leu and Na^+^ release.

### The *holo-occluded *state stabilized during the passage from OF to IF state secludes the substrate from both EC and IC environments

**Figure 3:**
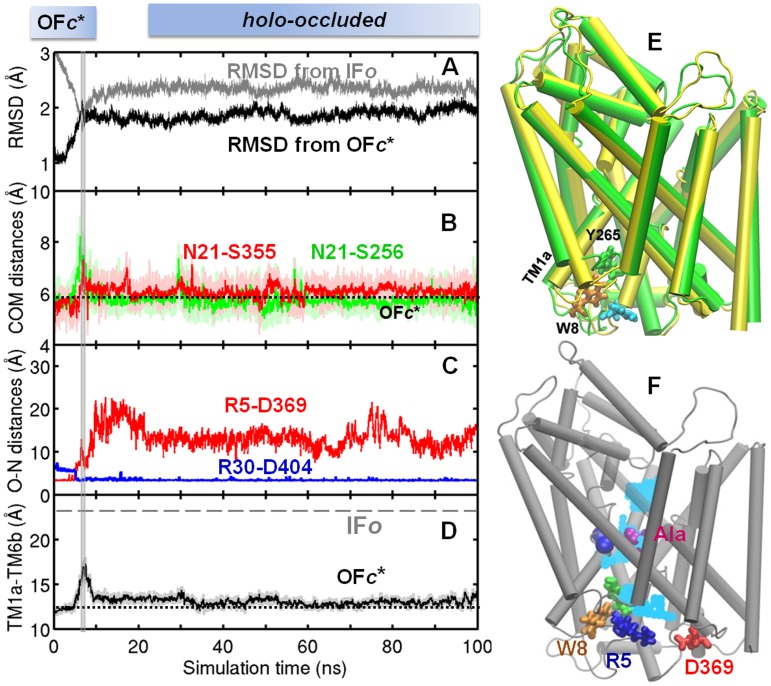
Passage to *holo-occluded* state, starting from OFc* state. Time evolutions are shown for (**A**) RMSD relative to IF*o* crystal structure (*gray*) and OFc* structure (*black*), based on C^α^-atoms (**B**) CoM distances between N21 and S256 (*green*) and N21 and S355 (*red*) indicating that these pairs retain their positions typical of OFc* state (*dotted line*). (**C**) oxygen-nitrogen distances of R5-D369 (*red*) and R30-D404 (*blue*) showing that the IC-facing (former) salt-bridge is broken, while that at the EC vestibule retains its closed state. (**D**) CoM distance between TM1a (R11-A22) and TM6b (F259-Y268), indicating that OFc* values are retained (*dashed horizontal line* refers to the IF*o* crystal structure). Gray vertical bar marks the switch from tMD (*run 3*) to cMD (*run 7*) (see **Table 1**). The upper abscissa boxes here and in similar figures indicate the prevalent conformational state at various stages of the simulations. (**E**) Superposition of the *holo-occluded state* reached in the two independent runs *6* (*green*) *and 7* (*yellow*); see results from *run 6* in **Figure S1**). (**F**) Hydration pattern of the *holo-occluded* state.

The *holo-occluded* state was consistently reached and stabilized in two independent runs (*runs 6* and *7*) that were performed to explore the departure from OFc* towards IF state. A snapshot from the tMD *run 3* was adopted as initial conformer in both cases, to allow for efficient sampling of the conformational space visited during this transition (**Table 1**). **Figure 3** and **Figure S1** present the results from these two independent runs. In both figures, the portions in the time range 0≤*t*≤6.8 ns delimited by the vertical gray bar refer to the initiating tMD, and the remaining portions (up to 100 ns) display the gradual stabilization of the *holo-occluded* state, indicated by the horizontal bar along the upper abscissa. The *holo-occluded* state, once reached, remained comparatively stable throughout the entire duration of the two simulations. The RMSD between the two equilibrated *holo-occluded* conformers (at the end of the two runs, structurally aligned in **Figure 3E**) was 1.4±0.3 Å (mainly due to differences at the N-termini; see below), in support of the close reproducibility of the *holo-occluded* structure.

The *holo-occluded* state exhibited intermediate features between the OFc* and IF*o* states, evidenced by an RMSD of 2.2±0.2 Å from both (**Figures 3A** **and** **S1A**) and interhelical packing characteristics (**Table 2**). It was minimally hydrated (**Figure 3F**), as opposed to the high level of hydrations of OF and IF states. Access to water from *both* sides was restricted by the closed gates R30-D404 (**Figures 3C** **and** **S1C**) and F253-Y108 on the EC side, and by the ternary interaction (W8-Y268-Y265) on the IC side (**Figure 3E**), in addition to a tighter packing of TM helices compared to OF/IF states. Center-of-mass (CoM) distances for the pairs TM1b-TM10 and TM6a-TM10 lining the EC vestibule decreased by ∼1 Å as compared to those of the equilibrated OFc*, and by 2–4 Å compared to OF*o* (**Table 2**); while TM1a-TM6b pair at the IC-facing region maintained their closed association, typical of OFc* state (**Figures 3D** **and** **S1D**). All these structural features ensured the seclusion of the substrate from both EC and IC media.

Alongside with these unique features, we noted that the IC salt-bridge R5-D369 was disrupted - typical of IFo* state (**Figures 3C, F** **and** **S1C**). The expansion of the IC vestibule, characteristic of IF state, did not start, however, until complete seclusion of substrate from the EC environment. We also noted the dissociation of the salt bridge E6-R375 in this intermediate (**Figure S1C**), signaling the predisposition to transition to IFo* state, as confirmed by unbiased runs below. The unique ability of the N-terminal segment to undergo various switches in salt-bridges which stabilize particular conformers will be further elaborated below.

### Substrate release is initiated by destabilization of binding site and influx of water, and enabled by outward tilting of TM1a and TM5

**Figure 4:**
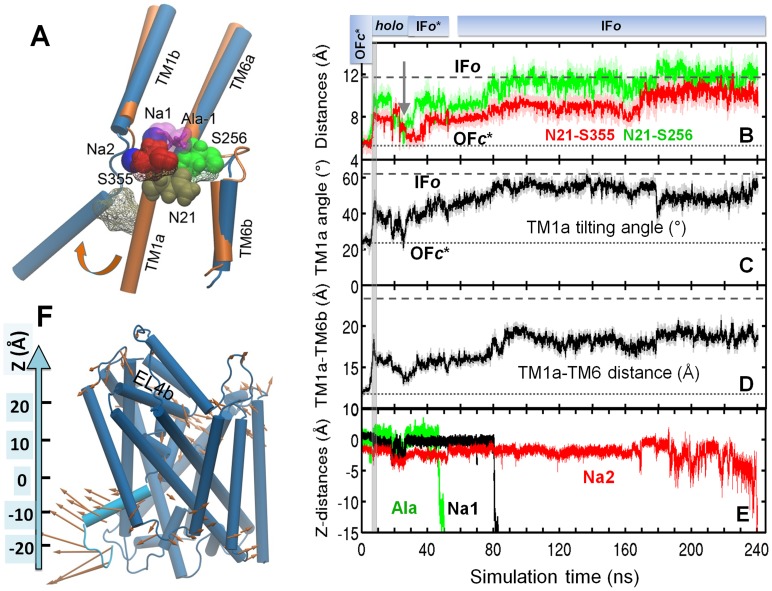
Time evolution of key molecular events during the passage from OF to IF state and release of substrate and Na^+^ ions. Release involves destabilization of interactions near N21 (*tan*) and TM1a tilting. (**A**) Alignment of TM1 and TM6 in the OF*o* (*orange*) and IF*o* (*blue*) crystal structures showing the reorientation of TM1a. N21, S256 (*green*) and S355 (*red*) are displayed in *vdW spheres* (OF*o*) and *wildframe* (IF*o*). Time evolutions of (**B**) N21-S256 (*green*) and N21-S355 (*red*) distances, based on residue mass centers; (**C**) TM1a tilting angle relative to the normal to membrane plane; (**D**) distance between TM1a (R11-A22) and TM6b (F259-Y268) residue mass centers; and (**E**) z-coordinates (see panel **F**) of Ala, Na1 and Na2, released at ∼50, 80 and 240 ns, respectively. (**F**) LeuT IF*o* conformation at 100 ns. TM1a is colored *cyan*. Arrows show the principal mode 1 deduced from essential dynamics analysis of cMD *run 8*. Gray vertical bar at 7.4 ns marks the switch from tMD (*run 3*) to cMD (*run 8*).

Next we examine the mechanism of substrate release. Substrate release was observed in five cMD simulations (*runs 8–11 and 18*). *Runs 8* and 9, initiated from the *holo-occluded* state, progressed to IFo* and then to IF*o* state. *Runs 10–11 and 18*, initiated from IFo* conformers from tMD *runs 3–5* (**Table 1**), progressed to IF*o*. The trajectories are illustrated in **Figures 4** (*run 8*), **S2** (*run 9*), **S3** (*run 10*) and **S4** (*run 11*). They all show consistent patterns, elucidating the molecular events that enable Ala:Na^+^ release and subsequent stabilization of a conformer that closely approximates the crystallographically resolved IF*o* structure, as described below. *Run 18* further proceeded to an *apo-occluded* state, and will be analyzed separately.

Destabilization of tight interactions at the Ala-binding pocket was a requirement for Ala release. The redistribution of N21 (TM1) interactions played a key role in initiating this local destabilization (**Figures 4A–B** **, S2A, S3B** and **S4B**). N21 intermittently formed hydrogen bonds with S256 (TM6) and S355 (TM8) prior to Ala binding. Disruption of these hydrogen bonds by Ala binding and influx of water molecules weakened the packing between TM1, TM6 and TM8, and initiated the reorientation of TM1a away from TM6b (**Figure 4C–D** and counterparts in **Figures S2–S4**). This reorientation was enabled by the existing kink in TM1 near N21. Note that the progression to IFo* was stalled (at *holo-occluded* state) when N21 maintained at least one of its inter-helical interactions (**Figures 3** and **S1**).

Complete release of substrate and cations was accomplished in all runs by cooperative switches that released IC-gating residues, along with increased TM1a reorientation (**Figures 4C and F** **and S2–S5**), up to ∼40° away from TM6b with respect to the OFc* configuration. In tandem, the increase in TM1a-TM6b distance at the IC face gradually exposed the IC vestibule. In addition, TM5 exhibited an outward tilting of 10–15° (**Figures S5**), which further weakened the packing of IC-exposed helices and favored IC water influx. The contribution of TM5 to mediating the transition from OF to IF conforms to that experimentally observed for LeuT-fold family members BetP [24] and MhP1 [25].

### IC gate opening is enabled by coupled redistribution of salt bridges involving N-terminal residues R5 and E6

The orientational flexibility of TM1a was essential to enable not only the IC vestibule opening, but also the N-terminal segment repositioning. The N-terminal segment was remarkably ‘active’ during the transition to IF state, practically swinging away from the IC vestibule toward the IC region (**Movies S1** and **S2**). This high mobility is consistent with a SERT model in which the N-terminus mechanics has been reported to be a requirement for action [10].

**Figure 5:**
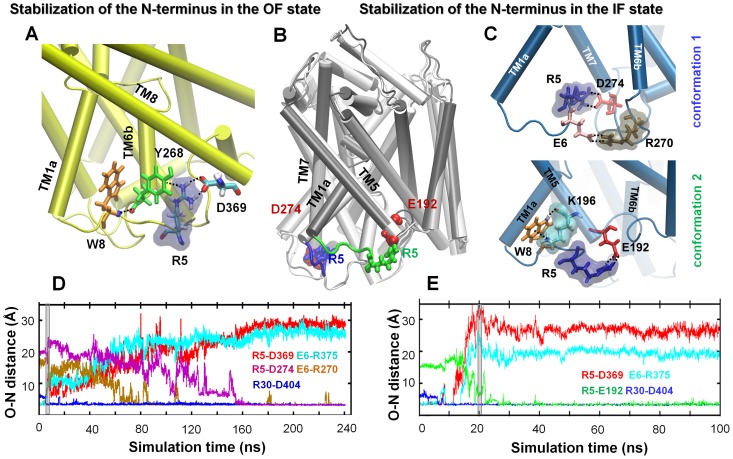
Involvement of N-terminal residues R5, E6 and W8 in the stabilization of LeuT OF and IF states. (**A**) A snapshot from cMD simulation of LeuT in the OF state, illustrating the cation-π interaction R5-Y268, the salt bridge R5-D369, and a hydrogen bond between W8 and Y268 backbones, which completely obstruct access to substrate-binding site from the IC region. (**B** and **C)** The same region in the IF state of LeuT. Two alternative N-terminal conformations, superimposed in (**B**) and further compared in (**C**) are observed for the IF*o* state reached at the end of *runs 8* (*white*) and *11* (*gray*): conformation 1 (**C**, *top*) stabilized by the salt bridge R270-D274 (TM7); and conformation 2 (**C**, *bottom*) stabilized by the salt-bridges R5-E192 and/or E6-R193 (not shown), and the cation-π interaction and hydrogen bond between W8 and K196. (**D** and **E**) Switches between salt-bridges involving R5 and E6 as the structure evolves from OFc* to IF*o* in the respective *runs 8* and *11*. The R5-D369 (*red*) and E6-R375 (*cyan*) salt bridges that close the IC vestibule in the OFc* give way to new salt bridges R5-D274 (*magenta*) and E6-R270 (*brown*) characteristic of IF*o* conformer 1 (**D**), or to R5-E192 (*green*) of IF*o* conformer 2 (**E**). The EC gate R30-D404 remains closed at all times during the transition OFc*→ *holo-occluded* →IFo* →IF*o*.

As mentioned earlier, a network of interactions between the N-terminus (R5 and W8) and TM helices TM6 (Y265 and Y268) and TM8 (D369) blocked the access of IC water to the substrate-binding pocket in the OF state prior to transition to IF state (**Figure 2A**), similar to the behavior observed in DAT [9]. **Figure 5** presents more details on these interactions and their time evolution. The diagrams compare the interactions involving the R5 and W8 in the OF (panel **A**) and IF (panels **B–C**) states. In the OF state, the pairs W8-Y268, R5-D369 and R5-Y268 form a tight network that completely block the access to the IC vestibule. The salt bridges R5-D369 and E6-R375 were also reported to restrict, if not prevent, the opening of the IC vestibule in an earlier tMD run [13]. In the IF state, R5 changes interaction partner, to either D274 (**C**, *top*), or E192 (**C**, *bottom*) on the respective helices TM7 and TM5 as described in the caption. Panel **B** shows the superposition of these two conformations where R5 and its sequential neighbors are colored *blue* or *green* (corresponding to the respective *top* and *bottom* diagram in **C**) These conformers were reproduced both by independent runs (*runs 8* and *18* for conformer 1; and *9* to *11* for 2), and further confirmed by additional cMD simulations (*runs 14–17*) performed to explore the conformational space near the IF*o* state.

**Figure 5** panels **D** and **E** display the time evolution of these interactions during the transition from IFc* to IF*o* state. The transition to IF*o* is marked by the rupture of the salt-bridges R5-D369 and E6-R375. In the meantime, the EC gating pairs R30-D404 and Y108-F253 (not shown) remained tightly associated, thus preventing the leakage of substrate or Na^+^ back to EC region, in line with alternate access mechanism.

As a further investigation of the conformational space accessible to the IF LeuT, we performed six additional cMD simulations of the IF*o* state (*runs 12*–*17*). The RMSDs from the IF*o* crystal structure remained around 1.4±0.3 Å in all six runs, and the TM1a helix exhibited wide open conformations as in the crystal (**Figure S6**). The N-terminus sampled both conformations 1 and 2 (**Figure 5B–C**) confirming the predisposition of R5 to form alternative salt bridges. Interestingly, the N-terminus also effectively prevented the penetration of lipids into the IC vestibule in the IF*o* state. In two test runs performed without the N-terminus (*runs 12* and *13*), the surrounding lipid molecules were observed to insert into the IC vestibule. No such insertions took place in the other runs (*runs 14–17*) performed with the intact N-terminal segment.

### IC pore for substrate/sodium release

**Figure 6:**
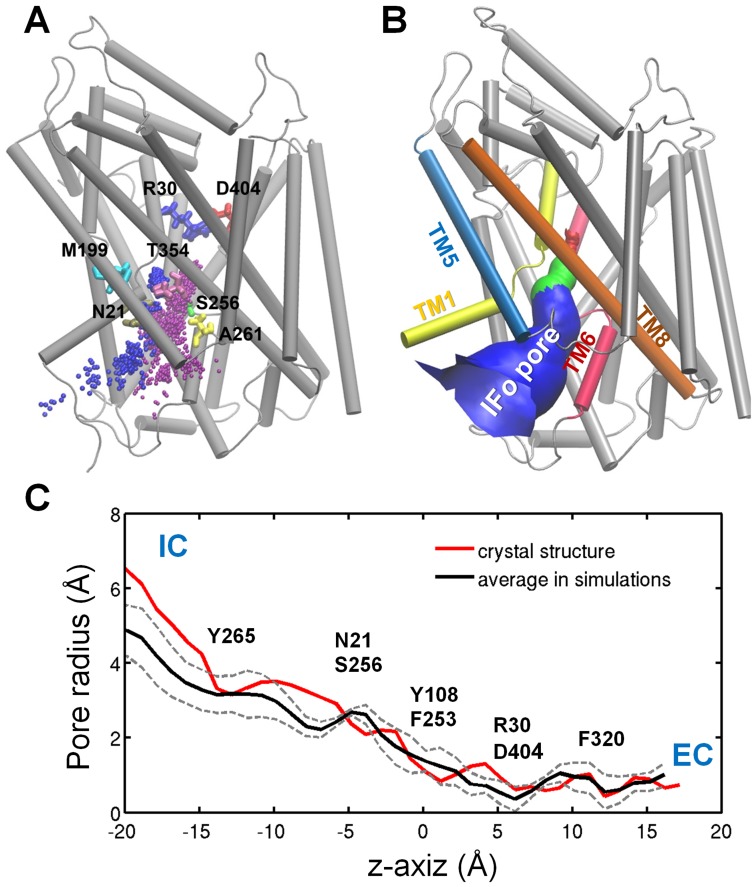
Substrate/cations release pathways and pores. IC pore observed in the IF*o* state reached in *runs 8–11*, and *18*, in accord with the pore detected in the IF*o* crystal structure. (**A**) Exit trajectories of Ala (*purple dots*) and Na^+^ ions (*blue dots*) observed in *run 18*; (**B**) IC pore depicted based on X-ray structure of IF*o*; and (**C**) Comparison of pore size profiles as a function of the elevation along the z-axis computed for MD equilibrated IF*o* conformers and the IF*o* X-ray structure (*red* curve). *Black* curve represents the average pore radii based on the IF*o* conformers in all five runs (*8*–*11*, and *18*). Dashed curves show the standard deviation.

In all five cMD simulations of substrate and cation release (*runs 8–11 and 18*), the substrate and cations were released through the IC pore identified in the IF*o* X-ray structure [6] (**Figure 6A–B**). The pore radius profiles observed in the MD-predicted IF*o* states closely reproduced that of the IF*o* crystal structure (**Figure 6C**), whose stability in the lipid environment was further confirmed by our additional cMD simulations (*runs 12*–*17*) (see **Figure S6**).

**Figure 7:**
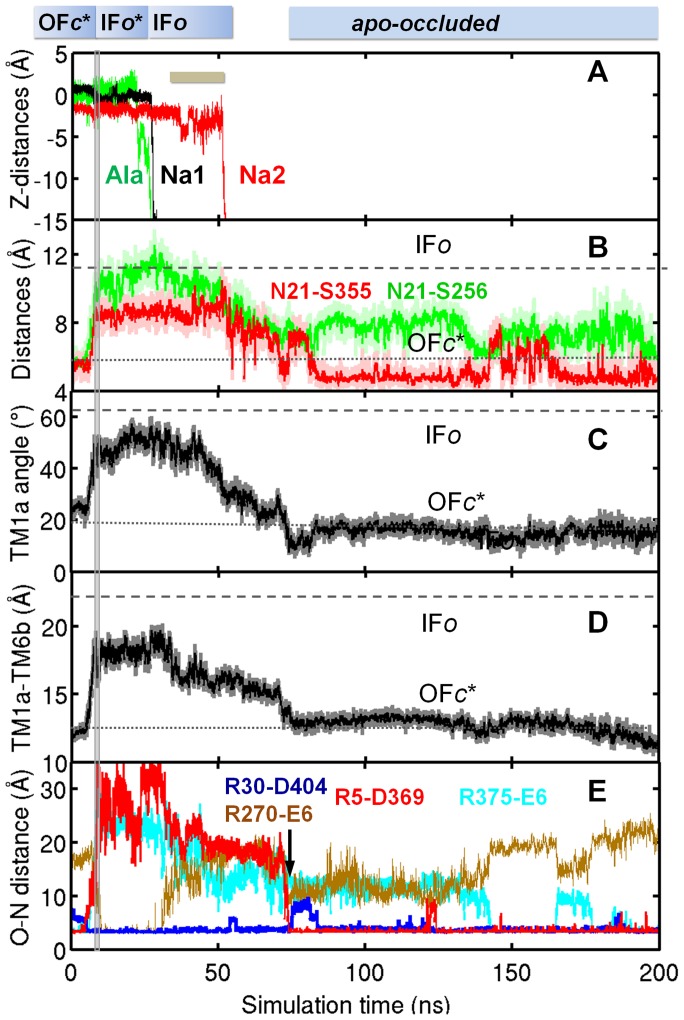
Complete release of substrate and Na^+^ ions and conformational change back to *apo-occluded* state, mediated by N-terminus. Time evolution of (**A**) the z-coordinates of Ala (*green*; released at ∼22 ns), Na1 (*black*; released at ∼26 ns), and Na2 (*red;* released at ∼52 ns); (**B**) N21-S256 (*green*) and N21-S355 (*red*) CoM distances; (**C**) TM1a tilting angle; (**D**) TM1a-TM6b distance; (**E**) N-O distances between salt-bridge forming/breaking pairs (labeled). Gray vertical bar at 8 ns marks the switch from tMD (*run 3*) to cMD (*run 18*).

While the path was consistently maintained, the order of releases showed some differences. In three of the five runs (*8, 9* and *18*) Ala was released first. This was succeeded by Na1 and then Na2 in both *runs 8* (**Figure 4**) and *18* (**Figure 7**), while the Na^+^ ions remained bound till the end (93 ns) of *run 9* (**Figure S2**). In the other two (*runs 11* and *10*; respective **Figures S4** and **S3**), Na2 was released either during, or immediately after, the preceding tMD runs (*4* and *5*, respectively), which led to almost simultaneous releases of Ala and Na1 in *run 10*, and Na1 followed by Ala in *run 11*. Given that the release events (and times) were completely independent of the preceding biased runs in the former three cases, it is likely that Ala is released first, succeeded by Na^+^ ions, although the occurrence of a different order cannot be ruled out. Regardless of the order, the same translocation pathway (IF*o* pore; **Figure 6B**) was reproduced (for both substrate and Na^+^ ions) in all runs.

### Spontaneous transition of the transporter back to OF state after substrate release, assisted by the N-terminal segment

In both *runs 18* and *19*, a transition back to an *apo-occluded* state was observed after the release of substrate and Na^+^ ions to the cytoplasm. **Figure 7** illustrates the successive events in *run 18*. This transition was facilitated by intermittent formation of hydrogen bonds between N21 and Y265, and between N21 and S355, which became gradually tighter, and returned to their OFc* values and thus sealing the substrate-binding (empty) site to block access from the IC environment (**Figure 7B**). Furthermore, TM1a underwent a reverse tilting (**Figure 7C**) toward its value in the OFc* structure, until it completely closed the IC vestibule by tight interaction with TM6b (**Figure 7D**).

Basically, after passage through an intermediate state partially occluded to the EC and IC regions (at 50–70 ns in **Figure 7**), the symporter settled in an *apo-occluded* conformer (**Figure 2**). Both the EC gate R30-D404 and IC gate R5-D369 are closed in this conformer (**Figure 7E**; **Movie S2**), thus preventing access of substrate/cation from the either region.

While these changes are suggestive of a transition toward the OFc* state, closer examination showed that the packing of key TM helices in the *apo-occluded* state differs from those in the OF (and IF) state: the EC-facing TM1b-TM10 and TM6a-TM10 are closer (than those in OF conformers) by 3.5–4 Å, and the IC-facing TM1a-TM6b pair is closer than its IF counterpart by about 8 Å (**Table 2**). Notably, the interhelical distances are comparable to those assumed in the *holo-occluded* state, and even tighter presumably due to the absence of substrate and cations that would otherwise occupy a space at the binding pocket.

**Figure 8:**
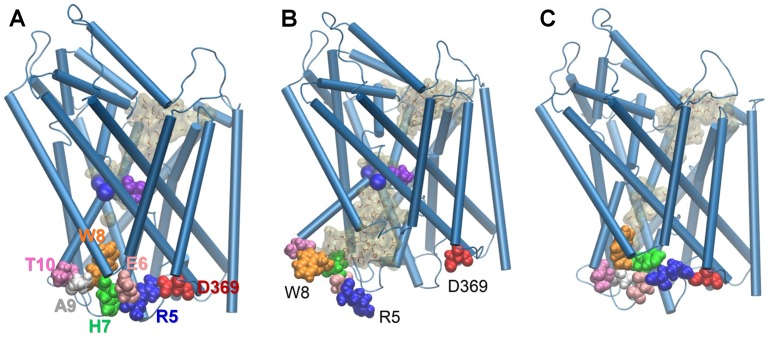
Regulation of substrate: Na^+^ release and transition back to *apo-occluded* state by redistribution of ineractions at LeuT N-terminus. (**A**) the initial conformer in the OFc* state; (**B**) a conformer representative of the IFo* state prior to substrate (Ala, *purple vdW*) and Na^+^ ions (*blue spheres*) release; and (**C**) *apo-occluded* state stabilized after the release of substrate and ions, close to the original OFc* state. R5 (*blue*), E6 (*pink*), H7 (*green*), W8 (*orange*), A9 (*white*), and T10 (*magenta*) are displayed in space-filling. Water molecules in the EC and IC vestibules are shown in semi-transparent *tan*. A–C display snapshots at 1 ns, 20 ns and 200 ns, from Figure 7.

N-terminal residues (R5-T10) played a significant role at this step of the transport cycle (**Figure 8**). Closure of the IC vestibule was enabled by re-formation of the salt-bridge R5-D369 consistently observed in *runs 18* and *19* (**Figures 7E** **and S7**), hydrophobic interactions between W8, L14, M18, and W63 (**Figure S8**), and hydrogen bond re-formation between W8 and Y265. The dissociation of the salt-bridge R270-E6 prompted the reorientation of TM1a to approach TM6b, and the formation of the salt bridge R5-D369 clearly drove TM1a to its closed position typical of OFc* state (**Figure 7C–E**). Upon closure of the IC gate, W8 penetrated into the IC vestibule (**Figure 8C**), minimizing the water occupancy therein. It is interesting to note intermittent breaking of the salt-bridge R30-D404 (**Figure 7E**), signaling the ensuing ability to open the EC gate.

All these observations provide firm evidence for the occurrence of a highly stable *apo-occluded state* prior to the transition of LeuT to OF*o* state to resume the transport cycle. No crystal structure has been resolved to date for LeuT in the *apo-occluded* state. However, betaine transporter (BetP), a structural homologue, has been newly crystallized in an *apo-occluded* state [24], which lends support to the possible stabilization of a similar state by LeuT.

## Discussion

### Major findings: Elucidation of two occluded structures (*apo* and *holo*) and the mechanism of substrate release

The present study, together with our previous simulations that focused on substrate/cation binding events [4], provides for the first time a complete mapping of the sequence of molecular events and structural changes that take place during the Na^+^-coupled substrate transport by LeuT. Our simulations reveal at atomic resolution the successive stages (**Figure 2**) from substrate recognition to closure of the EC gate upon substrate/cation binding [4], accompanying rearrangements of TM helices to proceed to a *holo-occluded* state, opening of the IC gate, release of substrate and ions, closure of IC gate, and transition back to a highly stable *apo-occluded* state which is proposed to precede the final transition to OF*o* state, to resume the transport cycle.

The *holo-occluded* and *apo-occluded* structures are newly identified here. They share many structural features, both on a local scale (closed EC and IC gates and N-terminal interactions) and global scale (same packing geometry between TM1, TM6 and TM10; **Table 2**).

The study also highlights the involvement of the N-terminal segment in stabilizing, if not regulating, functional transitions. The structure and dynamics of LeuT N-terminal segment have been elucidated for the first time.

Here is a summary of the observed mechanism of release succeeding substrate binding, reproduced in repeated runs, described in **Figures 2** **–** **8**: First, Ala binding stabilizes a *holo-occluded state* where both the EC and IC gates are closed, ensured by both local interactions and TM1-TM6-TM10 interhelical packing (**Table 2** **;** **Figure 3**). Destabilization of this structure starts near the Ala-bound broken (energetically frustrated) portions of helices TM1 and TM6, via disruption of interactions that N21 (TM1) makes with S256 (TM6) and S355 (TM8). The weakening of these interhelical interactions triggers (the pre-existing capability of) TM1a to reorient outward by up to ∼40° (**Figure 4**). TM1a reorientation is accompanied by a redistribution of interaction involving the N-terminal residues (e.g. disruption of salt bridges R5-D369 and E6-R375, and formation of others, R5-D274 or R5-E192) to expose the vestibule to the IC solution, while the EC gate R30-D404 remains closed at all times (**Figure 5**). The exposure of the IC vestibule precipitates an influx of IC water that further facilitates the dislocation of substrate and cations, all through the same pathway between TM1, TM5, TM6 and TM8 (**Figure 6**).

### Comparison with observations made for different members of LeuT fold family

The N-terminal residues of DAT (which shares the LeuT fold) have been pointed out to be implicated in the disruption of the OF state of DAT, and to exert a negative regulatory effect on DAT endocytosis [11]. It remains to be explored how the deletion of, or mutations in, this segment, that emerged here as a key regulator of functional rearrangements, drives even more drastic conformational changes conducive to endocytosis. The N-terminal segment in LeuT is significantly shorter than that in the eukaryotic homologue DAT (∼60 residues). The regulation of endocytosis presumably involves interactions with other regulatory proteins. Thus, the regulatory roles of the N-terminal segment in LeuT and DAT may differ in their mechanisms and implications.

Y265 and Y268 have been consistently observed in our simulations to form close interactions with W8 and thus contribute to regulating the IC vestibule closure as IC-gating residues that complement the pair R5-D369. Concerns have been raised [24] on the possible perturbation of the IC gate and TM1 mobility upon introduction of the mutation Y268A in the crystallization variant of LeuT IF*o* [6]. We restored the mutated residues back to their wild-type identities in our simulations. In all five cMD simulations (*runs 8 to 11* and *18*) of passage to IF state and release of substrate, the interactions holding TM1a (W8) and TM6b (Y265 or Y268) together broke before significant radial tilting of TM1a. The network of interactions between W8-Y265-W268 was maintained when the cycle was stalled at the *holo-occluded* state (**Figure 3E**). Inevitably, the orientation of TM1a is associated with the interactions between W8 and Y268. Therefore, it is conceivable that the Y268A mutation might have weakened the interaction between TM1a and TM6b, and thus shifting the equilibrium in favor of TM1a radial tilting away from TM6b. However, our study also indicates that the TM1a-TM6b interactions may be disrupted even in the absence of a mutation at this site, assisted by water influx. The observed water influx/efflux at various stages of transport is consistent with the transient formation of water-conducting conformers noted in membrane transporters [26].

Previous computational studies of LeuT-fold family members [13], [17], [18], [20] suggested that Na2 dissociates prior to the release of substrate, and triggers a cooperative transition to IF state. The present study showed variations in the order of cations release (**Figures 4**, **7**, **S3** and **S4**), which might be due to variations in the time-evolution of interactions between TM1a, TM6b, TM8, and TM5 along the release pore, as well as biases exerted in tMD runs. The tMD runs indeed tended to favor the release of Na2 first, whereas the cMD trajectories unbiased by tMD runs suggested the order Ala, Na1 and Na2. In LeuT, three glutamates (E112, E287, and E290) embedded in the central pocket may delay the release of Na^+^ ions in the IF state. We furthermore examined whether an Ala bound to a *secondary* binding site (S2) observed by Javitch and coworkers [27]–[30] could accelerate substrate release (from primary site S1). Our previous study supported the presence of site S2 the occupancy probability and binding affinity of which depends on the conformation of LeuT [4]. An Ala initially bound to S2 in *runs 8* and *18* remained bound in *run 8* while it escaped to the EC region in *run 18*. Correlated movements between the S2-bound Ala, and S1-bound Ala and Na2 were detected in the former case (**Figure S9**), consistent with previous observations [4]. However, no detectable acceleration in substrate/cation release was observed to be induced by these coupled movements.

The movements of TM1 undergone during the transition of LeuT from OF to IF state are comparable to those inferred from the comparison of LeuT crystal structures [6]; but are larger than those observed in other NSS family members such as BetP [24], MhP1 [25], and vSGLT [31]. Comparison of the crystal structures of LeuT, MhP1, vSGLT and BetP shows local structural differences near TM1a: in BetP, TM1a (R137-A148; counterpart of LeuT R11-A22) is connected to a long helical segment; but in LeuT, it is connected to a short disordered N-terminal tail (R5 to T10), and therefore enjoys higher conformational flexibility. Furthermore, the IC gating interactions, R5-D369 and W8-Y268, proposed for LeuT [6] and their counterparts in eukaryotic family members [9], [11], [32], [33] are not conserved among other NSS family members such as BetP, Mhp1 and vSGLT. Taken together, even though LeuT, MhP1 and BetP share similar architecture and robust mechanisms of alternating access between OF to IF states, the distinct redistributions of local inter-residue interactions near TM1a and N-terminal segment may be important in conferring their substrate specificity.

### Limitations of current computations

The time scale of transport is of the order of milliseconds to seconds. Even with the most advanced computational hardware and software, cMD simulation of secondary transporters in the presence of explicit membrane and water molecules cannot be extended beyond microseconds [8], [12]. The tMD and aMD techniques adopted in *runs 3–6* are approximations, aimed at accelerating events that are otherwise beyond the reach of cMD. The former may artificially drive reconfigurations into unphysical conformers especially if the spring constants adopted in the forces applied on the molecule are too stiff and if the run is performed for extended durations. To avoid such situations, we applied soft forces to the backbone only, for short durations, followed by long unbiased cMD simulations that allowed the transporter to relax and sample energetically favorable conformers, consistent with the procedures adopted in previous work [4]. Likewise, aMD runs allow for fast isomerization of side chains and overall accelerated dynamics, which may lead to a drift towards conformations that may not be naturally accessible, if performed for extended durations. In both cases, the reproducibility of the results and their physical realism were examined by multiple runs and/or comparison with relevant experimental and computational studies.

### Future work: Testable hypotheses for further investigation

The present study provides a number of testable hypotheses on the role or interactions of particular residues at various stages of the transport cycle. For example, no progression from *holo-occluded* to IF state was observed as long as the N21-S256 and N21-S355 interactions were maintained, while their disruption was a key step in prompting TM1a opening and ensuing transition IF state. Cross-linking experiments with cysteines substituted at those positions may provide further evidence on the involvement of these interactions in enabling the functional transitions. Likewise, breaking of the salt-bridges R5-D369 and E6-R375 is pivotal for exposing the IC vestibule, while plugging of W8 in the opening via interactions with Y265 and Y268 is key to occluding the same gate following substrate release. Transition back to the *apo-occluded* is enabled by restoring the salt-bridge R5-D369 that ‘seals’ the gate and pulls back TM1a closer to TM6b. It remains to be seen if substitution of alanines, for example, at those key positions could reduce, if not obstruct, substrate uptake or current flow.

We also noted that R5 and E6 adopt different orientations and form alternative salt-bridges with residues from TM7 (E274 and R270) or TM5 (E192 and R193) in the populating two alternative conformations in the IF state. Site-directed mutagenesis experiments with double mutants E192A and R193A, or E274A and R270A may help confirm the functional relevance of these particular helices (and salt bridges) in the regulation of the transport cycle.

Much attention has been given to the conformational flexibility of TM1 as the structural element that undergoes the most dramatic change between open and closed states of the IC-vestibule, but this study also draws attention to TM5 (and TM7), the IF*o* pore opening role of which may be interrogated by site-directed mutagenesis and electrophysiology experiments.

## Methods

### Simulation systems and processes

Atomic MD simulation systems corresponding to OFc* (**Figure 1A**; PDB: 2A65) [5] and IF*o* (**Figure 1B**; PDB: 3TT3) [6] states of LeuT were prepared using VMD [34], following our previous approach [4]. Briefly, the missing loops were re-constructed and refined using MODELLER 9.10 [35]; and the substituted/mutated residues were restored back to their wild type amino acids. The protonation states of titratable residues were assigned based on pKa calculations performed in ref [36]. In particular, E112, E287 and E419 were neutralized. Then the transmembrane (TM) domain was inserted into the center of a pre-equilibrated and solvated POPC membrane. Fully equilibrated TIP3 waters and 0.1 M NaCl were added to neutralize the system in a simulation box of 100×100×96 Å^3^. Unless otherwise stated, all simulation systems contained a LeuT monomer (R5 to R507), two Ala substrates, 30 Na^+^, 35 Cl^−^, 212 POPC, and about 16,770 water molecules to add up to ∼86,900 atoms.

Multiple MD runs, including aMD [22], tMD [13] and cMD were carried out, building on our earlier study of LeuT [4], summarized in **Table 1**. The simulation techniques and protocols are described below in some details. The runs consist of five sets, each comprising at least two independent runs.

#### 1. Conformational fluctuations near the OFc* state

Two cMD runs (*runs 1* and *2*) were performed for LeuT OFc* state (**Figure 1A**). The system was first energy minimized for 20,000 steps, followed by an equilibration of 2 ns during which the backbone constraints (of 10 kcal/(mol.Å^2^)) on LeuT were gradually removed. Unrestrained Nosé-Hoover [37], [38] constant pressure (P = 1 bar) and temperature (T = 310 K) (NPT) simulations were continued for 30 ns. The C^α^ RMSD from the OFc* crystal structure reached a plateau of 1.3±0.2 Å after 10 ns in both runs.

#### 2. Initiation of the transition away from OFc* toward IF*o*

Three tMD runs (*runs 3–5*) were carried out to trigger the transition from OFc* to IF*o* state, using equilibrated OFc* conformations as initial structures [4]. Targeted forces were applied to backbone atoms of G13- R507 (residues R5-T10 are not resolved in the IF*o* crystal stucture). The tMD runs were performed to initiate the induction of conformational transitions that may lead to substrate release, similar to previous work performed for other transporters (see for example ref [39]).

#### 3. Transition OFc* → *holo-occluded* (*runs 6–7*)

One aMD and one cMD run starting from the 6.8 ns snapshot of *run 3* were performed for 94 ns. We chose as initial state this particular snapshot because it represented a conformer where both the EC and IC gates were temporarily closed, and it permitted us to thoroughly investigate the dynamics of LeuT in the vicinity of this conformer in the absence of any biases.

#### 4. Substrate release (*runs 8–11* and *18*) and transition to *apo-occluded* state (*runs 18* and *19*)

*Run 8* and *run 9* were initiated from the 7.4 ns snapshot of *run 3*, which was used as a representative *holo-occluded* conformer. 233 ns MD in *run 8* permitted us to visualize the consecutive transitions from *holo-occluded* to IFo*, and from IFo* to IF*o*, with the help of conventional simulations. In *run 9*, similar structural transitions were observed which lead to the release of substrate within 93 ns cMD. *Runs 10–11* and *18* were initiated from conformers representative of IFo* state (see **Table 1**) and performed for 91 ns, 80 ns and 192 ns, respectively. They permitted us to investigate the fluctuations near the IFo* state, the release of substrate/ions and the conformational fluctuations near the IF*o* state. An external potential of −0.1 kcal/(mol.Å) was applied along the transmembrane direction between 22 ns and 44 ns (marked as a tan horizontal bar in **Figure 7A**) in *run* 18, to facilitate Na2 release. After the release of substrate and Na^+^ ions, the transporter spontaneously reconfigured into the *apo-occluded* state. The same transition was confirmed by *run 19* initiated 1 ns after substrate and sodium releases in *run 18*.

#### 5. Equilibrium dynamics of the IF*o* state with focus on the N-terminal segment (*runs 12–17*)

Three sets of 30 ns simulations, all in duplicate, were performed, the former was in the absence of N-terminal residues R5-T10, using the IF*o* crystal structure (PDB: 3TT3) where the N-terminal segment was not resolved, and the other two with two alternative N-terminal conformations identified in simulations: (*conformations 1* and *2*, **Figure 5C**). The protein structures were embedded into equilibrated and solvated POPC lipids. **Figure 1B** illustrates the starting IF*o* conformation, which contained LeuT, 189 POPC, 30 Na^+^, 35 Cl^−^, and about 16,700 water molecules to add up to about 86,000 atoms.

### Molecular dynamics (MD) simulation parameters and protocol

CHARMM36 force field with CMAP corrections was used [40]–[42] with NAMD package [43], following previous simulation protocol [4]. In aMD simulation, dihedral angle (*φ* rotations were accelerated by adding a boost potential Δ*V*  =  (*E* – *V*(*φ*))^2^/(*α* + *E* – *V*(*φ*)) to the original potential *V*(*φ*), whenever *V*(*φ*) fell below a threshold value *E* [22]. *E* and α were set to be 18,600 kcal/mol and 210 kcal/mol, respectively [4]. For tMD, a steering force of the form *F_tMD_*  =  ½ (*k/N*) [(RMSD(*t*) – RMSD*(*t*)] was applied, with the spring constant *k* = 200 kcal/(mol.Å^2^) [4]; *N* is the number of targeted atoms, RMSD(*t*) is the instantaneous departure from the target crystal structure, and RMSD*(*t*) is the target based on a linear decay from RMSD(*0*) to zero.

Simulation protocols included periodic boundary conditions, water wrapping, hydrogen atoms constrained via SHAKE, and evaluation of long-range electrostatic forces via the Particle Mesh Ewald (PME) algorithm [44]. The bonded and short-range non-bonded interactions were calculated at every time-step (2 fs), and electrostatic interactions were calculated every 4 fs. The cutoff distance for non-bonded interactions was 12 Å. A smoothing function was employed for the van der Waals (vdW) interactions at a distance of 10 Å. The non-bonded interactions list was updated every 20 time-steps for pairs within 13.5 Å.

### Trajectory analysis

VMD [34] with in-house scripts was used to analyze the structural and dynamical features of the systems, such as the RMSD, helical tilting angles, CoM distances, hydrogen bonds, and salt bridges. TM1a helical tilting was estimated either relative to the membrane normal, or TM1a orientation in the OFc* state. Principle component analysis (PCA) of MD trajectories was performed using ProDy [45]. The first mode displayed by arrows in **Figure 4F** accounts for 28% of the overall dynamics in *run 8*. The lowest frequency modes based on the anisotropic network model [46] were calculated using ProDy [45] and visualized using Normal Mode Wizard implemented in VMD. The Hessian matrix was built using all C^α^-atoms and a pairwise interaction cutoff of 15 Å. vdW and electrostatic interactions were calculated using the pairInteraction module implemented in NAMD [43]. The cavity size of the substrate binding pocket was calculated using the POVME algorithm [47]. Briefly, LeuT without any bound substrate was used for calculation. A grid encompassing the entire binding pocket was generated with 1.0-Å spacing. The grid points not occluded by protein atoms and connected through a series of adjacent grid points to the center of the binding site were used for calculating cavity volume. The pore size of the IC vestibule was calculated using HOLE [48] and visualized using VMD [34].

## Supporting Information

Figure S1 **Passage from OF state to** ***holo-occluded*** **state.** Time evolution of (**A**) RMSDs of the protein C^α^ atoms relative to IF*o* (*gray*) and OFc* (*black*) structures; (**B**) CoM distances of N21-S256 (*green*) and N21-S355 (*red*); (**C**) oxygen-nitrogen distances of R5-D369 (*red*), R375-E6 (*cyan*) and R30-D404 (*blue*); and (**D**) CoM distance for TM1a-TM6b. *Dotted* and *dashed* horizontal lines refer to values in the OFc* and IF*o* crystal structures, respectively. *Gray* vertical bar at 6.8 ns marks the switch from tMD (*run 3*) to aMD (*run 6*) (see **Table 1**).
(TIF)

Figure S2 **Passage from the OF to IF state.** Time evolution of (**A**) N21-S256 (*green*) and N21-S355 (*red*) distances, based on residue mass centers; (**B**) TM1a tilting angle relative to the normal to membrane plane; (**C**) distance between TM1a and TM6b (F259-Y268 mass centers); (**D**) oxygen-nitrogen distances of R5-D369 (*red*), E6-R375 (*cyan*), R5-E192 (*green*), and R30-D404 (*blue*); (**E**) z-coordinates of Ala, Na1 and Na2; and (**F**) RMSD of the protein (based on C^α^-atoms) from the IF*o* crystal structure. Gray vertical bar at 7.4 ns marks the switch from tMD (*run 3*) to cMD (*run 9*).
(TIF)

Figure S3 **Release of substrate and cations and stabilization of IF** ***o*** **state.** Time evolution of (**A**) the z-coordinates of Ala (*green;* released at ∼10 ns), Na1 (*black*; released at ∼10 ns), and Na2 (*red*; released at ∼10 ns); (**B**) CoM distance between N21 and S256 (*green*) and between N21and S355 (*red*); (**C**) TM1a tilting angle relative to the membrane normal; (**D**) the distance between TM1a and TM6b segments; (**E**) oxygen-nitrogen distances of R5-D369 (*red*), R375-E6 (*cyan*), R5-E192 (*green*), R193-E6 (*tan*), and R30-D404 (*blue*); and (**F**) RMSD of the protein (based on C^α^ atoms) from the IF*o* crystal structure. Gray vertical bar at 9 ns marks the switch from tMD (*run 5*) to cMD (*run 10*) (see **Table 1**).
(TIF)

Figure S4 **Passage to IF** ***o*** **state, starting from the OF** ***c*** * state. Time evolution of (**A**) the z-coordinates of Ala (*green;* released at ∼40 ns), Na1 (*black;* released at ∼20 ns), and Na2 (*red*; released at ∼15 ns); (**B**) the distances of N21-S256 (*green*) and N21-S355 (*red*); (**C**) TM1a tilting angle relative to the membrane normal; (**D**) the distance between TM1a and TM6b; (**E**) oxygen-nitrogen distances of R5-D369 (*red*), E6-R375 (*cyan*), R5-E192 (*green*), and R30-D404 (*blue*); and (**F**) RMSD of the protein C^α^ atoms relative to IF*o* crystal structure (PDB: 3TT3). Gray vertical bar at 20 ns marks the switch from tMD (*run 4*) to cMD (*run 11*).
(TIF)

Figure S5 **TM5 outward tilting coupled with TM1a tilting facilitates the formation of the IC vestibule for release of substrate/sodium ions.** Comparison of the ∼50 ns snapshot in *run 8* (*silver cylinder*) when Ala was released (see **Figure 4**) with the OFc* crystal structure (*white transparent cylinder*; PDB: 2A65), highlighting the reorientations of TM1a and TM5. TM1a, TM6b and TM5 are shown in *solid blue* (simulation) and *transparent blue* (PDB: 2A65).
(TIF)

Figure S6 **Stability of the IF** ***o*** **conformer and the open conformation of TM1a in the IF** ***o*** **state.** The IF*o* crystal structure remains stable in the lipid environment (**A**), and its TM1a segment exhibits wide open conformations as seen in the crystal structure (**B**). (**A**) Time evolution of LeuT C^α^ RMSD with respect to the IF*o* crystal structure (PDB: 3TT3). *Red*, *pink*, *blue*, *cyan, black* and *gray* curves display the results from the respective *runs 12-17* (**Table 1**); and (**B**) CoM distances between TM1a and TM6b.
(TIF)

Figure S7 **Closure of the EC gate R5-D369 observed during the transition to the** ***apo-occluded*** **state from the IF state.** Trajectories from *runs 18 and 19* are shown, after the complete release of substrate and cations. In both simulations, the putative IC gate residue R5 moved over 15 Å and reformed the salt-bridge R5-D369. The *apo-occluded* state reached in the two independent runs share the same structural features (RMSD  = 1.3±0.3 Å).
(TIF)

Figure S8 **Hydrophobic interactions between W8 and L14, M18 and W63 facilitate the closure of the W8 intracellular gate.** Snapshots from (**A**) 10 ns (**B**) 40 ns and (**C**) 92 ns of *run 18*.
(TIF)

Figure S9 **Z-direction (along the membrane) distance between the S1 site and the instantaneous CoM positions of primary Ala (** ***green*** **; released around 50 ns), Na1 (** ***black*** **, released at 80 ns), Na2 (** ***red*** **; released at 240 ns) and secondary Ala (** ***blue*** **; S2); Results refer to 233 ns cMD (** ***run 8*** **), preceded by 7.4 ns tMD (** ***run 3*** **) (** **Table 1** **and** **Figure 4** **).** The secondary Ala (*blue*) exhibited downward movements toward the IC region (see arrows), correlated with the movements of the primary substrate and Na2.
(TIF)

Movie S1 **Sequence of events during the passage from OF** c* **to IF** ***o*** **state, including the passage over the** ***holo-occluded*** **state.** Trajectories refer to 240 ns simulations shown in **Figure 4** (tMD *run 3* followed cMD *run 8*) (see **Table 1** and **Figures 4** and **5D**). The EC-gating residues R30, D404, Y108, and F253, are shown in *blue, red, green* and *orange* sticks (*upper*), respectively. The putative four IC gate residues R5, D369, Y265, and W8, are colored *blue, red, green* and *orange* sticks (*lower*), respectively. Ala is shown as *purple* VDW representation. Na1 and Na2 are displayed in *blue* spheres. The segment composed of N-terminal and TM1 residues R5-A22 is colored *cyan* and the TM6b helical segment (F259-Y268) in *yellow*. Ala release takes place around ∼50, and those of Na1 and Na2 at ∼80 and 240 ns, respectively.
(MPG)

Movie S2 **Release of substrate and Na^+^ ions and conformational transition to** ***apo-occluded*** **state, starting from IF** ***o*** * state. Trajectories refer to 192 ns cMD *run 18*. Note the close interactions between the putative IC gate residues (*lower*) W8 (*orange*)-Y265 (*green*) and R5 (*blue*)-D369 (*red*) succeeding the release of substrate and cations, and the ensuing reorientation of TM1a (*cyan*), all of which stabilize the *apo-occluded state*.
(MPG)
