# Supplementary figures and images for: Complete Mapping of Substrate Translocation Highlights the Role of LeuT N-terminal Segment in Regulating Transport Cycle
Source: PLoS Comput Biol. 2014 Oct 9;10(10):e1003879. doi: 10.1371/journal.pcbi.1003879 (PMC4191883; doi:10.1371/journal.pcbi.1003879)

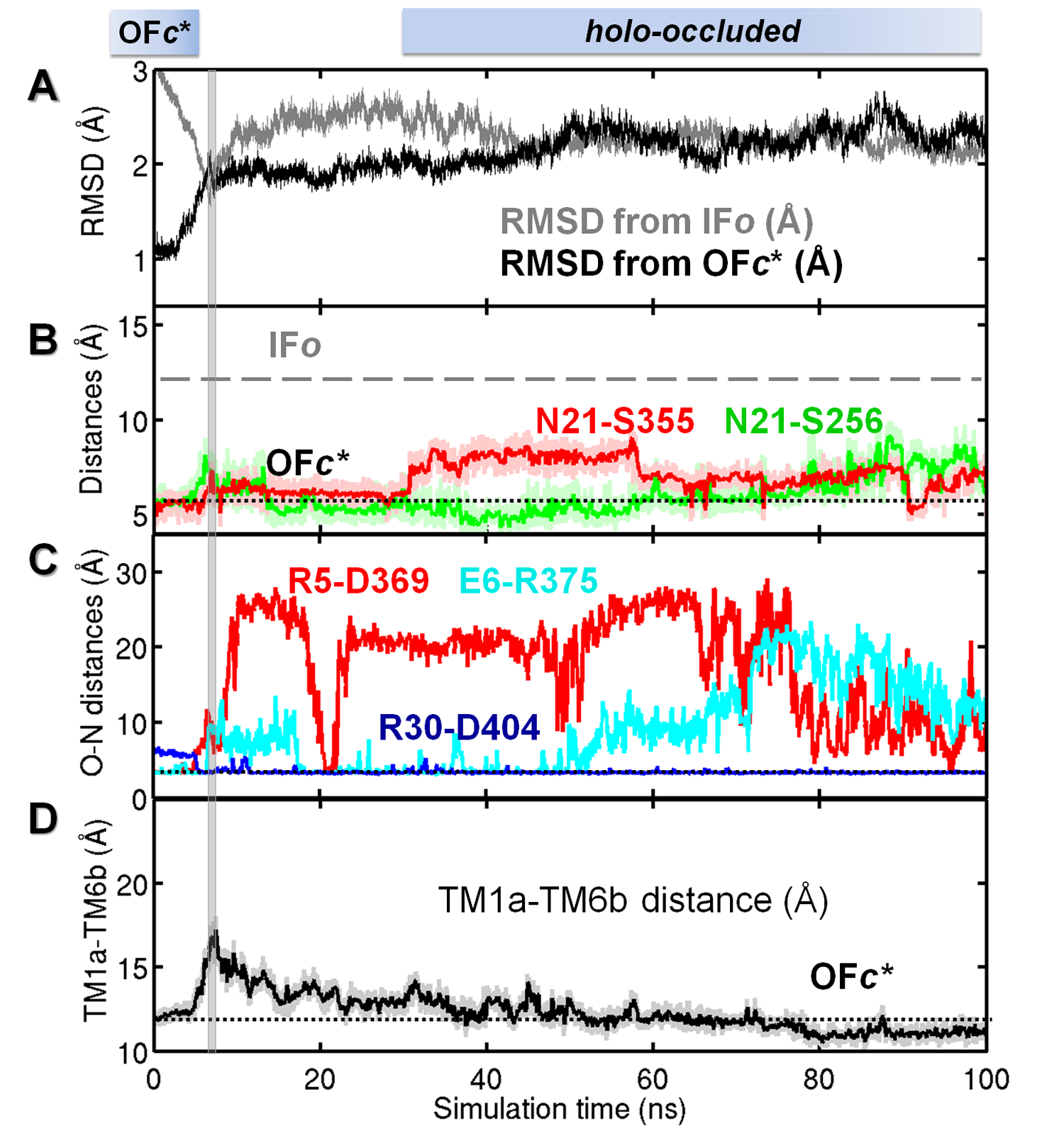

Supplement: Figure S1 — Passage from OF state to holo-occluded state. Time evolution of (A) RMSDs of the protein Cα atoms relative to IFo (gray) and OFc* (black) structures; (B) CoM distances of N21-S256 (green) and N21-S355 (red); (C) oxygen-nitrogen distances of R5-D369 (red), R375-E6 (cyan) and R30-D404 (blue); and (D) CoM distance for TM1a-TM6b. Dotted and dashed horizontal lines refer to values in the OFc* and IFo crystal structures, respectively. Gray vertical bar at 6.8 ns marks the switch from tMD (run 3) to aMD (run 6) (see Table 1 ). (TIF) [file pcbi.1003879.s001.tif]

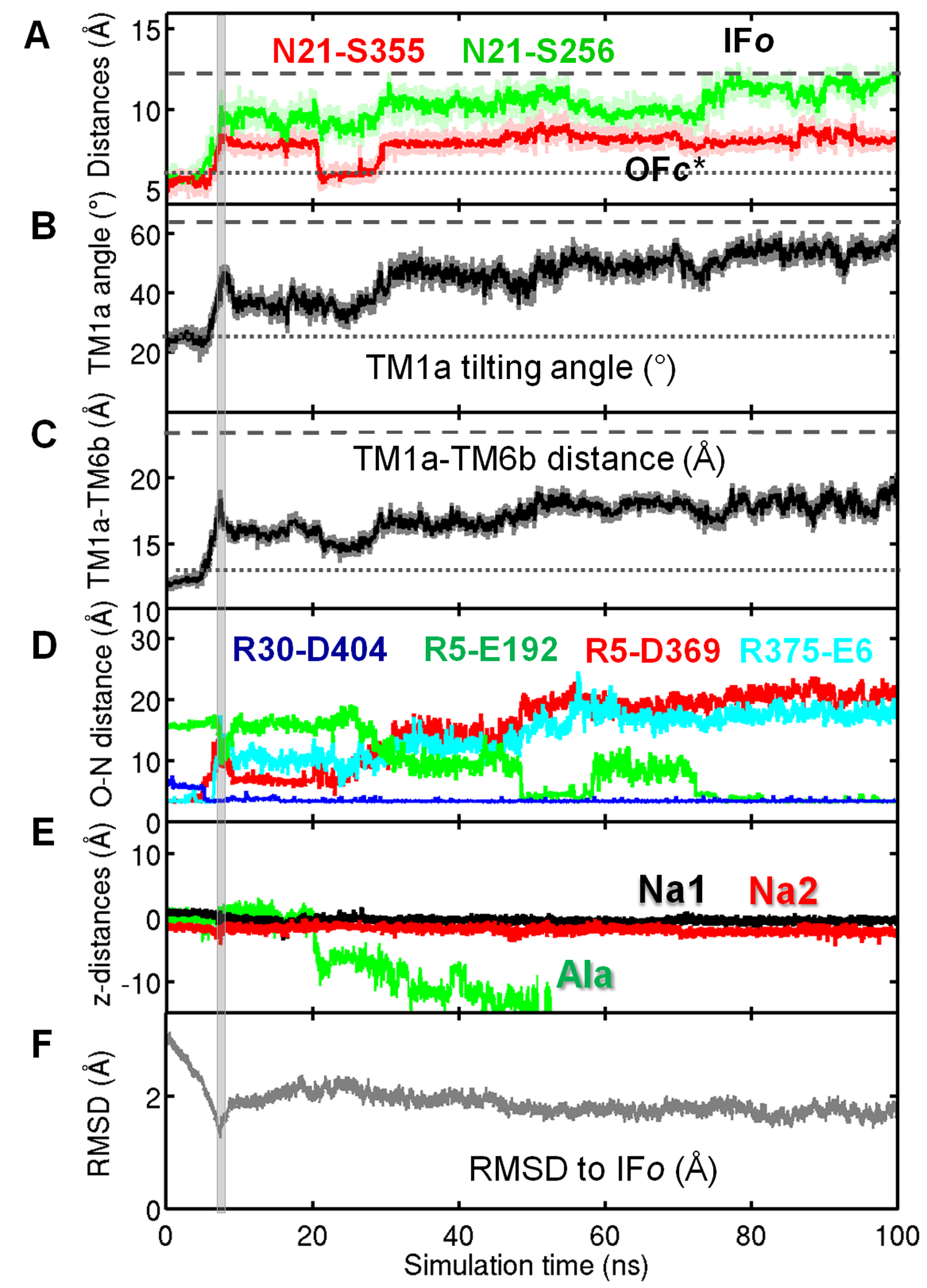

Supplement: Figure S2 — Passage from the OF to IF state. Time evolution of (A) N21-S256 (green) and N21-S355 (red) distances, based on residue mass centers; (B) TM1a tilting angle relative to the normal to membrane plane; (C) distance between TM1a and TM6b (F259-Y268 mass centers); (D) oxygen-nitrogen distances of R5-D369 (red), E6-R375 (cyan), R5-E192 (green), and R30-D404 (blue); (E) z-coordinates of Ala, Na1 and Na2; and (F) RMSD of the protein (based on Cα-atoms) from the IFo crystal structure. Gray vertical bar at 7.4 ns marks the switch from tMD (run 3) to cMD (run 9). (TIF) [file pcbi.1003879.s002.tif]

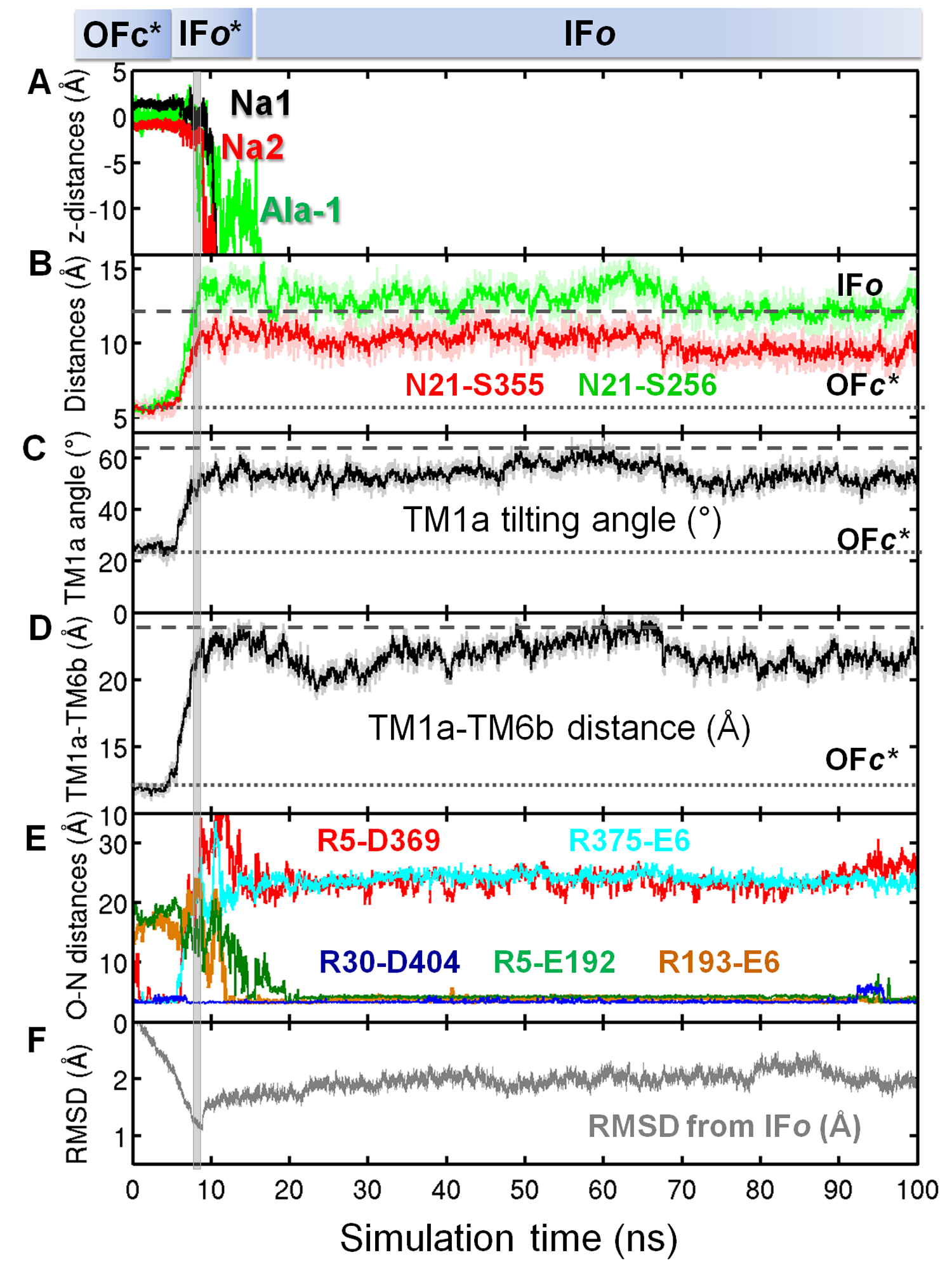

Supplement: Figure S3 — Release of substrate and cations and stabilization of IF o state. Time evolution of (A) the z-coordinates of Ala (green; released at ∼10 ns), Na1 (black; released at ∼10 ns), and Na2 (red; released at ∼10 ns); (B) CoM distance between N21 and S256 (green) and between N21and S355 (red); (C) TM1a tilting angle relative to the membrane normal; (D) the distance between TM1a and TM6b segments; (E) oxygen-nitrogen distances of R5-D369 (red), R375-E6 (cyan), R5-E192 (green), R193-E6 (tan), and R30-D404 (blue); and (F) RMSD of the protein (based on Cα atoms) from the IFo crystal structure. Gray vertical bar at 9 ns marks the switch from tMD (run 5) to cMD (run 10) (see Table 1 ). (TIF) [file pcbi.1003879.s003.tif]

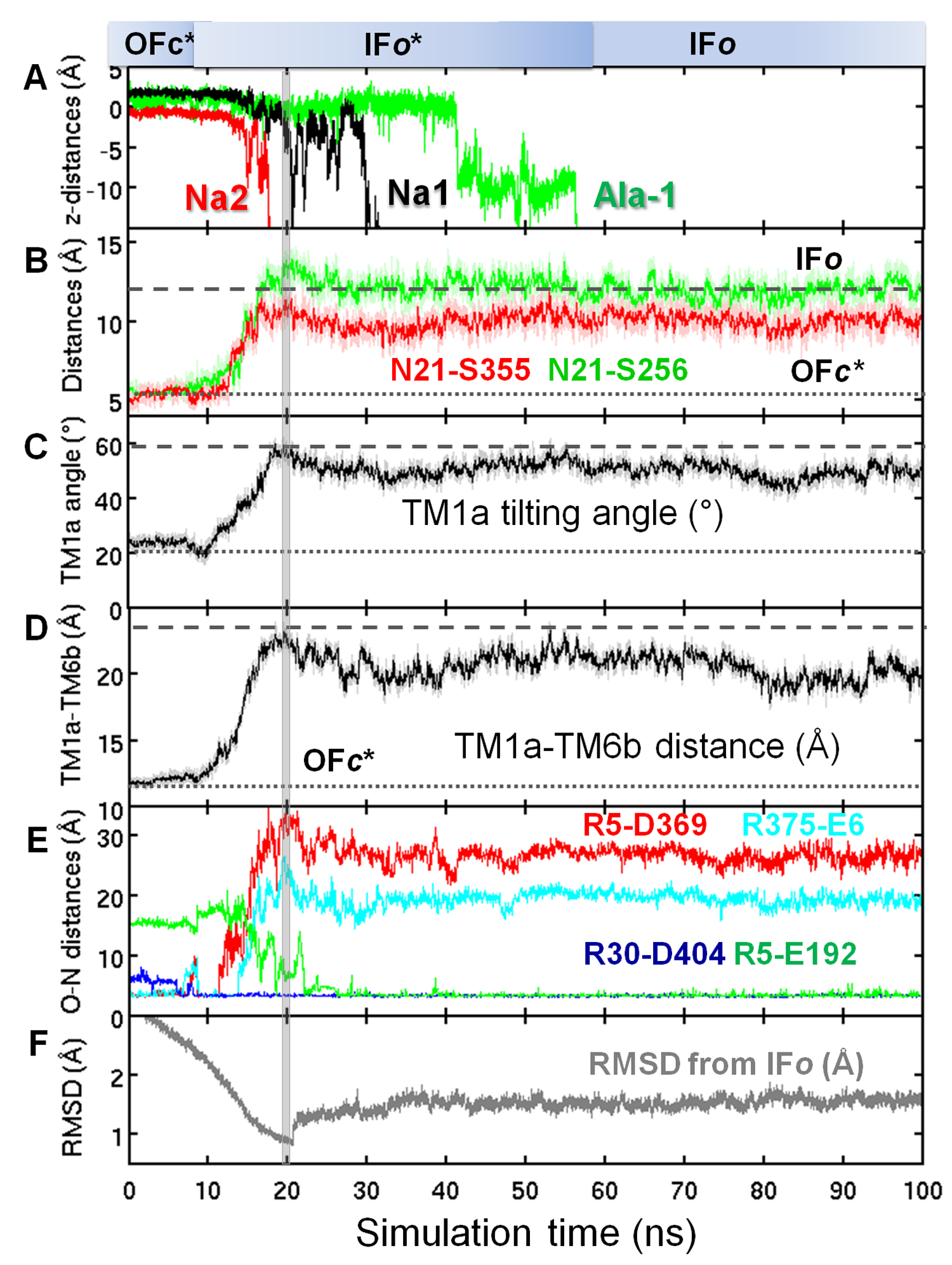

Supplement: Figure S4 — Passage to IF o state, starting from the OF c * state. Time evolution of (A) the z-coordinates of Ala (green; released at ∼40 ns), Na1 (black; released at ∼20 ns), and Na2 (red; released at ∼15 ns); (B) the distances of N21-S256 (green) and N21-S355 (red); (C) TM1a tilting angle relative to the membrane normal; (D) the distance between TM1a and TM6b; (E) oxygen-nitrogen distances of R5-D369 (red), E6-R375 (cyan), R5-E192 (green), and R30-D404 (blue); and (F) RMSD of the protein Cα atoms relative to IFo crystal structure (PDB: 3TT3). Gray vertical bar at 20 ns marks the switch from tMD (run 4) to cMD (run 11). (TIF) [file pcbi.1003879.s004.tif]

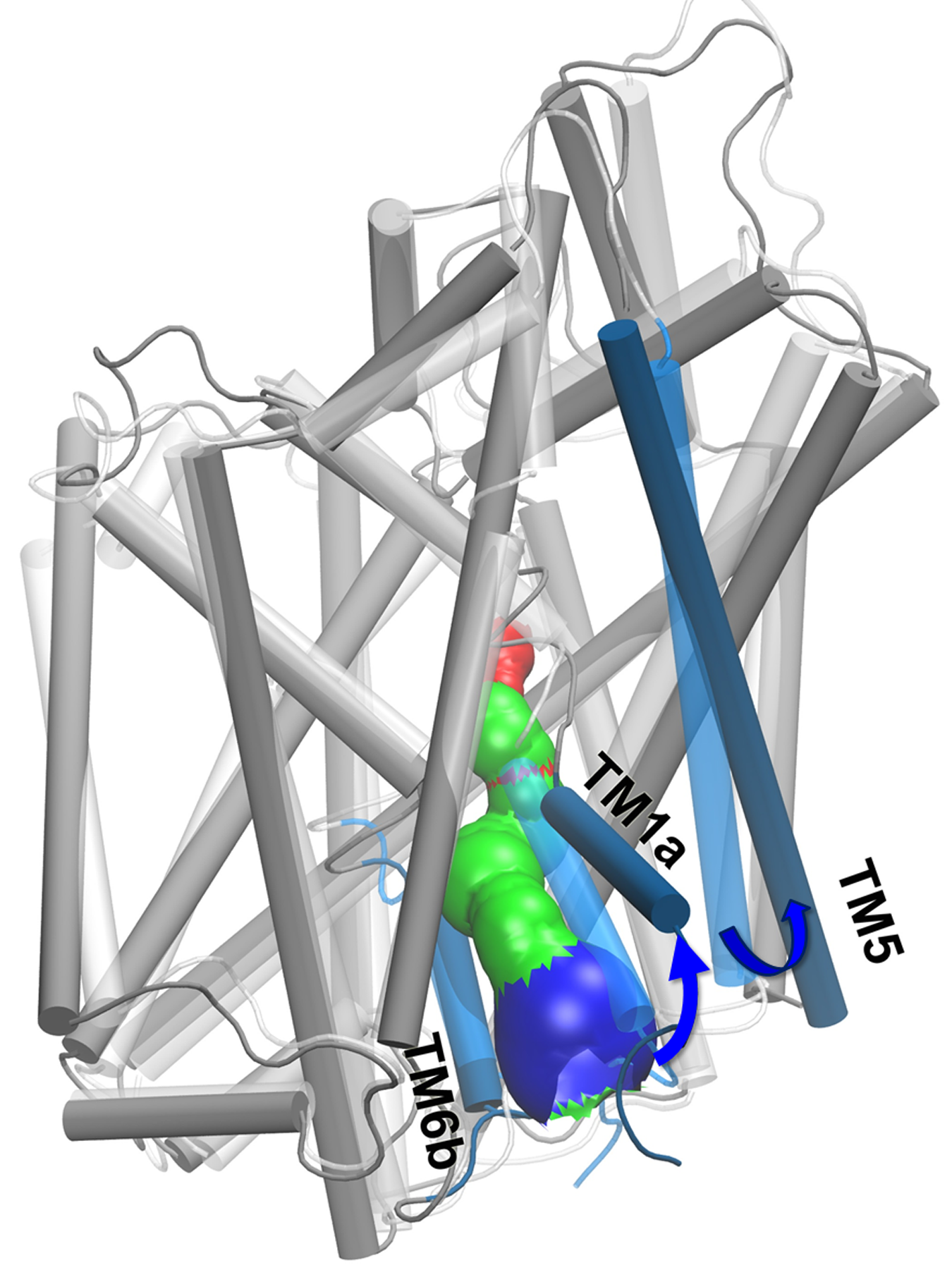

Supplement: Figure S5 — TM5 outward tilting coupled with TM1a tilting facilitates the formation of the IC vestibule for release of substrate/sodium ions. Comparison of the ∼50 ns snapshot in run 8 (silver cylinder) when Ala was released (see Figure 4 ) with the OFc* crystal structure (white transparent cylinder; PDB: 2A65), highlighting the reorientations of TM1a and TM5. TM1a, TM6b and TM5 are shown in solid blue (simulation) and transparent blue (PDB: 2A65). (TIF) [file pcbi.1003879.s005.tif]

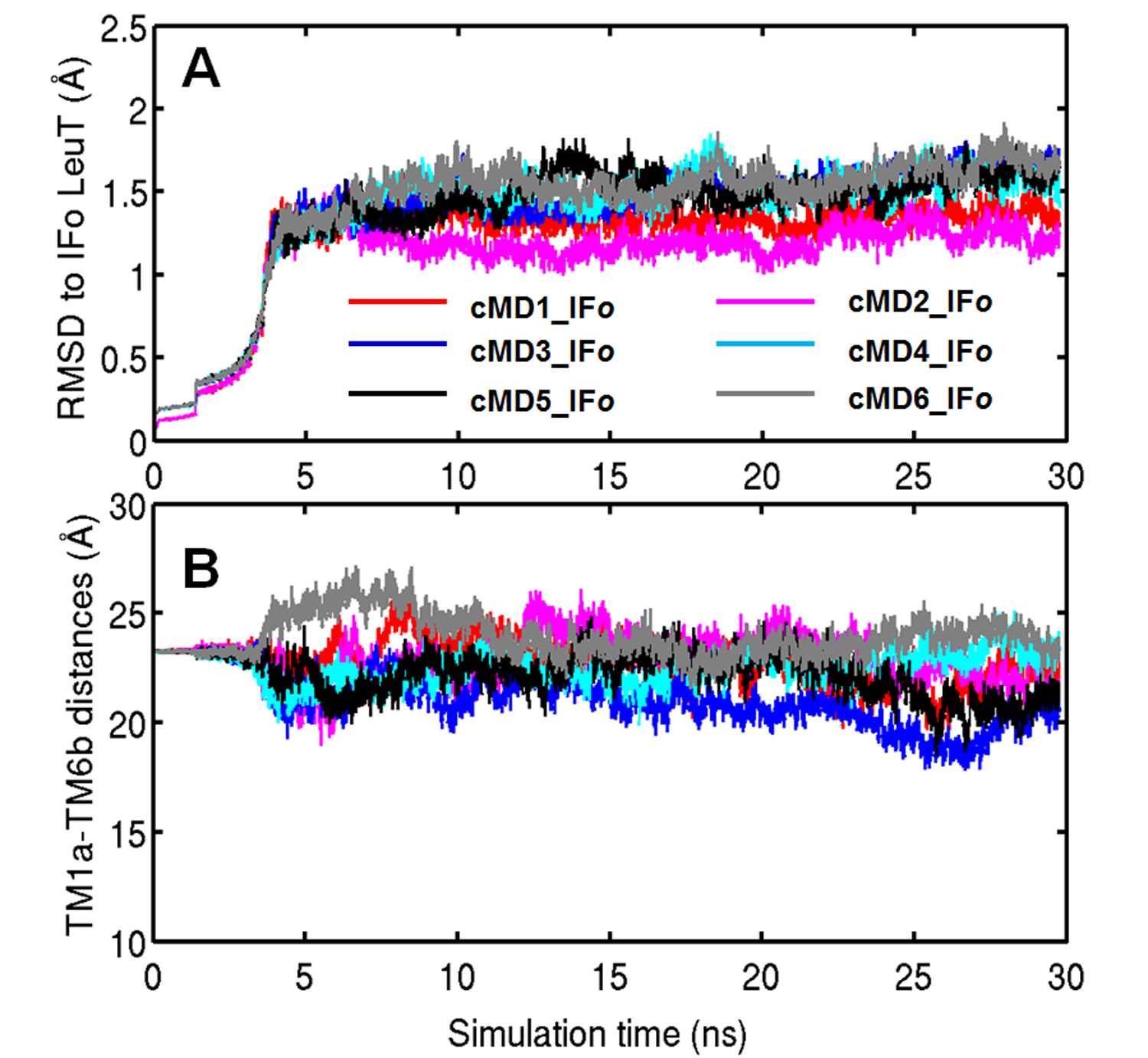

Supplement: Figure S6 — Stability of the IF o conformer and the open conformation of TM1a in the IF o state. The IFo crystal structure remains stable in the lipid environment (A), and its TM1a segment exhibits wide open conformations as seen in the crystal structure (B). (A) Time evolution of LeuT Cα RMSD with respect to the IFo crystal structure (PDB: 3TT3). Red, pink, blue, cyan, black and gray curves display the results from the respective runs 12-17 ( Table 1 ); and (B) CoM distances between TM1a and TM6b. (TIF) [file pcbi.1003879.s006.tif]

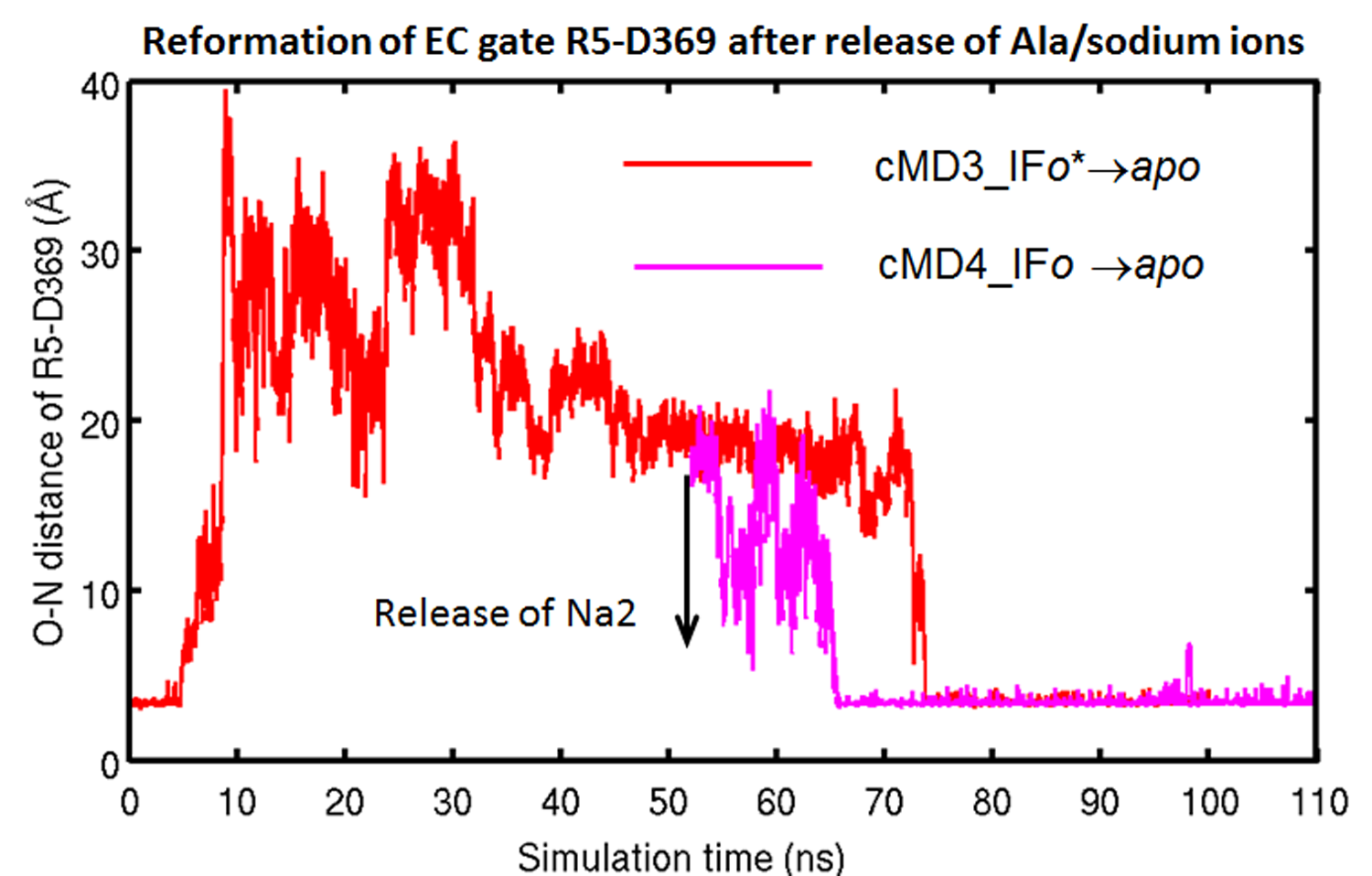

Supplement: Figure S7 — Closure of the EC gate R5-D369 observed during the transition to the apo-occluded state from the IF state. Trajectories from runs 18 and 19 are shown, after the complete release of substrate and cations. In both simulations, the putative IC gate residue R5 moved over 15 Å and reformed the salt-bridge R5-D369. The apo-occluded state reached in the two independent runs share the same structural features (RMSD = 1.3±0.3 Å). (TIF) [file pcbi.1003879.s007.tif]

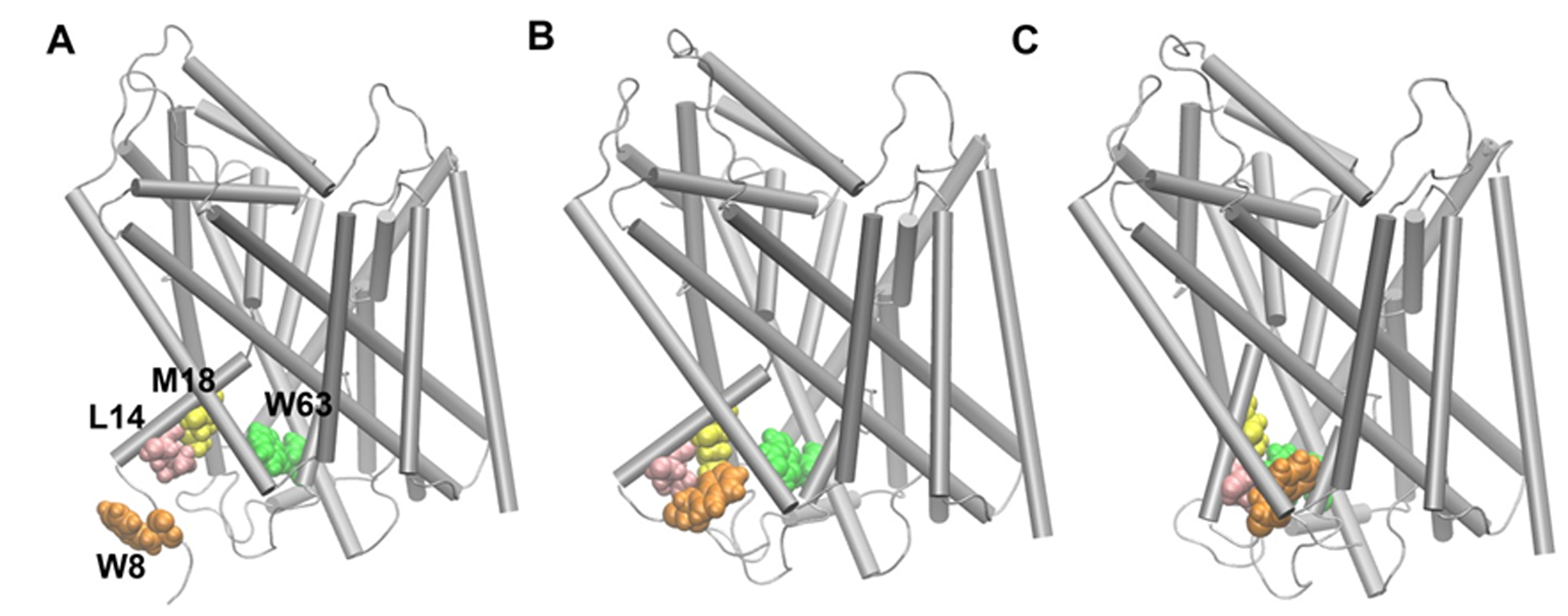

Supplement: Figure S8 — Hydrophobic interactions between W8 and L14, M18 and W63 facilitate the closure of the W8 intracellular gate. Snapshots from (A) 10 ns (B) 40 ns and (C) 92 ns of run 18. (TIF) [file pcbi.1003879.s008.tif]

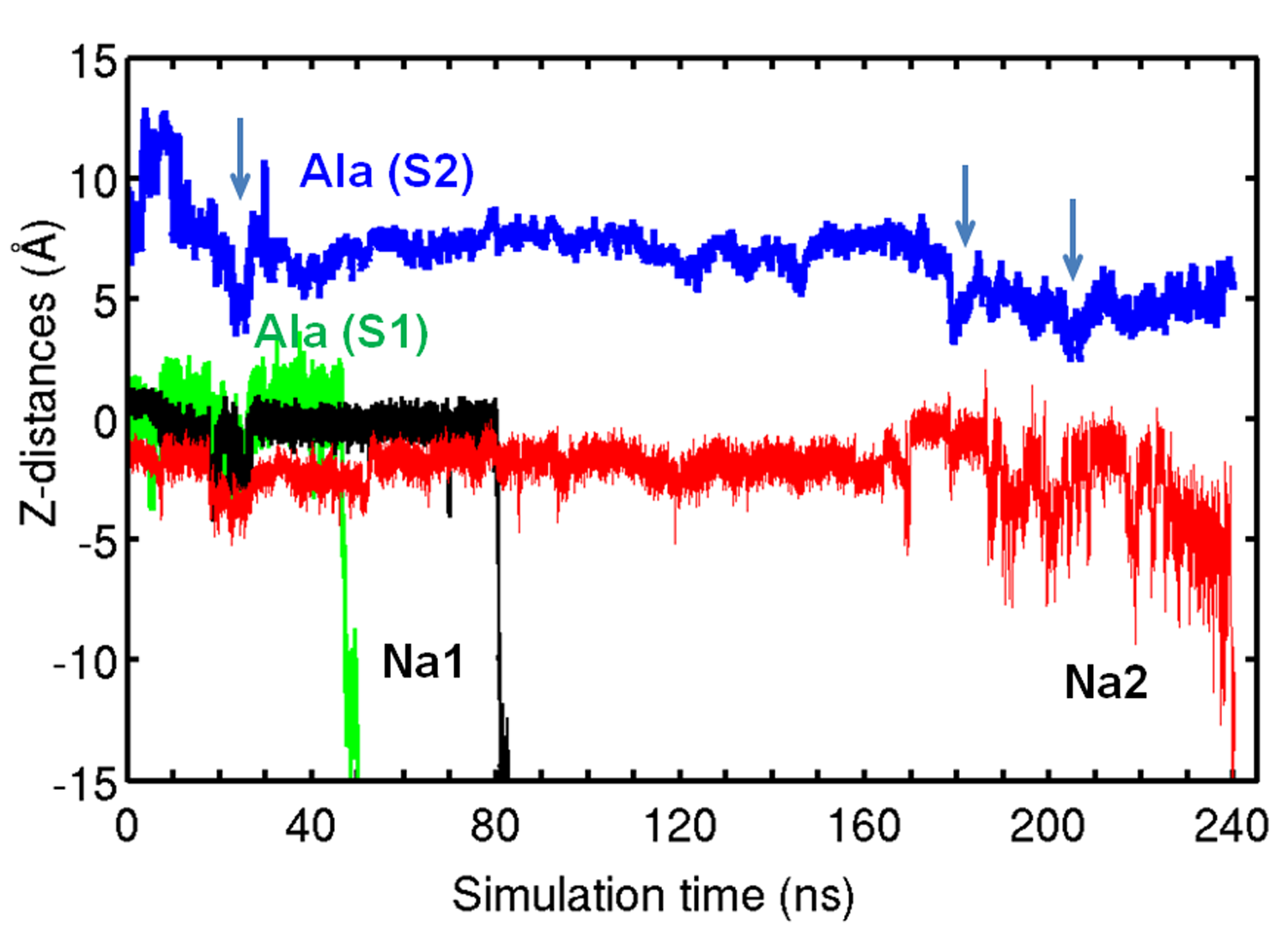

Supplement: Figure S9 — Z-direction (along the membrane) distance between the S1 site and the instantaneous CoM positions of primary Ala ( green ; released around 50 ns), Na1 ( black , released at 80 ns), Na2 ( red ; released at 240 ns) and secondary Ala ( blue ; S2); Results refer to 233 ns cMD ( run 8 ), preceded by 7.4 ns tMD ( run 3 ) ( Table 1 and Figure 4 ). The secondary Ala (blue) exhibited downward movements toward the IC region (see arrows), correlated with the movements of the primary substrate and Na2. (TIF) [file pcbi.1003879.s009.tif]
